# Transdermal administration of herbal essential oil alleviates high-fat diet-induced obesity by regulating metabolism and gut microbiota

**DOI:** 10.3389/fphar.2025.1565030

**Published:** 2025-03-19

**Authors:** Zu-Wen Ye, Qi-Yue Yang, Dong-Hua Yang, Qiao-Hong Lin, Xiao-Xia Liu, Feng-Qin Li, Fang-Fang Yan, Ping Luo, Si Qin, Fang Wang

**Affiliations:** 1 Cancer Research Center, The Jiangxi Province Key Laboratory for Diagnosis, Treatment, and Rehabilitation of Cancer in Chinese Medicine, Jiangxi University of Chinese Medicine, Nanchang, China; 2 Department of Endocrinology, Hospital of Chengdu University of Traditional Chinese Medicine, Chengdu, China; 3 New York College of Traditional Chinese Medicine, Minola, NY, United States; 4 Lab of Food Function and Nutrigenomics, College of Food Science and Technology, Hunan Agricultural University, Changsha, China

**Keywords:** obesity, essential oil, lipid mechanism, inflammation, gut microbiota

## Abstract

**Introduction:**

Obesity, a global health challenge, is characterized by excessive fat accumulation and associated metabolic disorders. The ZhiZhu decoction, a traditional Chinese herbal formula consisting of *Citrus aurantium* L. (ZS, ZhiShi in Chinese) and *Atractylodes macrocephala Koidz* (BZ, Baizhu in Chinese), is widely recognized in clinics for its gastrointestinal regulatory effects.

**Methods:**

The chemical composition of ZS-BZ essential oil (ZBEO) was characterized using gas chromatography-mass spectrometry (GC-MS). Concurrently, we conducted *in vitro* investigations using HepG2 hepatoma cells to evaluate its anti-lipid deposition potential. To further elucidate the anti-obesity mechanisms, an *in vivo* model was established through high-fat diet (HFD)-induced obese rats, followed by transdermal ZBEO administration. Systemic analyses were performed integrating serum metabolomic profiling via UPLC-QTOF-MS and gut microbiota dynamics assessment through 16S rRNA gene sequencing.

**Results:**

ZBEO, rich in atractylon, D-limonene, and γ-elemene and shown to reduce lipid accumulation. Transdermal ZBEO administration in obese rats led to significant weight loss and improved serum metabolic indexes related to the POMC/CART signaling pathway. Additionally, ZBEO altered gut microbiota, enhancing beneficial bacteria and affecting metabolic pathways linked to obesity.

**Discussion:**

We discovered that ZBEO exerts a significant influence on obesity by modulating key biological processes, including glucose metabolism, lipid metabolism, and the composition of gut microbiota.

## 1 Introduction

Obesity is a chronic and progressive metabolic disorder, particularly on dysregulated carbohydrate and lipid metabolism. Obesity is characterized by significant overweight, excessive fat accumulation, and energy imbalance (Hruby and Hu, 2015; De Lorenzo et al., 2019). Since 1975, the global prevalence of obesity has rapidly increased, severely impacting physical and mental health of human beings. Obesity is a major risk factor for type 2 diabetes (T2DM), dyslipidemia, hypertension, cardiovascular diseases, non-alcoholic fatty liver disease, and colorectal cancer (Marteau et al., 2009; Saltiel and Olefsky, 2017). Its etiology is complex, involving disturbances in gastrointestinal hormone levels (Aukan et al., 2023), lipid and energy metabolism (Tang et al., 2023), hypothalamic feeding regulation (Sousa-Ferreira et al., 2014), and gut microbiota dysbiosis (Brahe et al., 2016). Current treatments include medicinal therapy, which can cause side effects such as gastrointestinal discomfort, muscle loss, and dependence, as well as surgical treatment which may cause serious complications (Ahn et al., 2024). Therefore, seeking safe and effective novel drugs that can regulate metabolism is of great significance for the clinical prevention of obesity and its related diseases.

Traditional Chinese Medicine (TCM) offers a promising perspective for treating complex and chronic diseases owing to its “multi-components, multi-targets and multi-pathways pharmacological characteristics”. In view of the multifactorial etiology of obesity, a single drug often fails to achieve comprehensive therapeutic efficacy; hence, a synergistic polypharmacy strategy becomes attractive. For example, Huang et al. found that the famous TCM formula Ling Gui Zhu Gan can reduce serum levels of triglycerides (TG), total cholesterol (TC), low-density lipoprotein cholesterol (LDL-c), body mass index (BMI), and body weight, and increase high-density lipoprotein cholesterol (HDL-c). It affects inflammation and lipid digestion pathways to alleviate or reverse hyperlipidemia and obesity (Huang et al., 2022). In addition, Katherin et al. found that *Lippia alba* essential oil can reverse adipocyte hypertrophy by affecting the viability, lipid mobilization, and adipogenesis of adipocytes *in vitro* (Bonilla-Carvajal et al., 2022). Furthermore, Gioxari et al. showed that *Chios mastiha* essential oil alleviates metabolic abnormalities by regulating inflammation and antioxidant processes in individuals with abdominal obesity and metabolic abnormalities (Gioxari et al., 2023).

The formula “Zhizhu Fang”, first recorded in the “Jin Kui Yao Lue”, is a famous prescription for “regulating the spleen and stomach and ameliorating glucose and lipid metabolism”. *Citrus aurantium L.* (ZS, ZhiShi in Chinese) can be used for coronary heart disease (Nie et al., 2022), internal stagnation, and phlegm stagnation, with effects such as promoting gastrointestinal motility, anti-cancer (Wang L. et al., 2022), regulating lipid metabolism (Jia et al., 2016; Guo et al., 2019), and antioxidant effects (Liu et al., 2017). The essential oil of ZS (Wang et al., 2020), primarily composed of D-limonene, has antibacterial, anti-inflammatory, and anti-cancer effects (Su et al., 2020). Similarly, *Atractylodes macrocephala Koidz* (BZ, Baizhu in Chinese) can strengthen the spleen, dry dampness, and promote diuresis. Its essential oil contains atractylon and atractylenolide. Atractylon has the effect of diuretic, anti-inflammatory (Chen et al., 2016), anti-cancer, hepatoprotective (Kiso et al., 1983), blood pressure-lowering, blood sugar-lowering, anti-bacteria (Wang et al., 2019), anti-viral, and immune-regulating effects (Zhu et al., 2018). These findings suggest that BZ may have anti-obesity effects, making it valuable for potential applications in obesity treatment.

## 2 Materials and methods

### 2.1 Materials and reagents

ZS-BZ essential oil (ZBEO) was obtained from Changshengyuan Medicinal Plant Development (Mianyang, China). Orlistat was obtained from Zhongmei Huadong (H30019728, Hangzhou, China). Oleic acid was sourced from Aladdin Industrial Corporation (Shanghai, China). Additionally, atractylon, D-limonene, and γ-elemene were obtained from Chengdu Pufeide Biological Technology Co., Ltd. (Chengdu, China). Enzyme-linked immunosorbent assay (ELISA) kits of rat tumor necrosis factor-α (TNF-α), interleukin-6 (IL-6), interleukin-17 (IL-17), rat adiponectin, rat visfatin, rat ghrelin, rat leptin, rat orexins A, rat glucagon-like peptide-1 (GLP-1), rat cholecystokinin (CCK), and rat aquaporin 7 (AQP7) were obtained from CUSABIO (E11987r, E04640r, E07451r, E11987r, E08941r, E09816r, E07433r, E08860r, E08117r, E08114r, E05124r, Wuhan, China).

### 2.2 GC-MS analysis on ZBEO

ZBEO was analyzed by gas chromatography-mass spectrometry (GC-MS) (Agilent, United States, Model 7890A/5975C). The sample was separated by an Agilent DB-624 (0.25 mm × 30 mm, 0.25 μm) capillary column. 100 μL ZBEO was diluted with n-hexane into 10 mL and then filtered through a 0.22 μm membrane before injection. One microliter of the diluted sample was injected in pulsed splitless mode at 250°C. Helium was used as the carrier gas at a constant flow rate of 1 mL/min. The GC oven temperature program was as follows: held at 40°C for 1 min, then increased to 220°C at a rate of 40°C/min, followed by a ramp to 280°C at 10°C/min, and held for an additional 5 min. For mass spectrometry, electron ionization (EI) was utilized with an electron energy of 70 eV, an ion source temperature of 230°C, a quadrupole temperature of 150°C, and an interface temperature of 250°C. The solvent delay was set to 3.0 min, and the mass scanning range was from m/z 30–650 amu. Relative quantification was performed using area normalization with three replicates. Peak identities were confirmed by matching mass spectra against the NIST 11 library, with a similarity threshold of ≥85%. Additionally, retention times were compared with those of authentic standards to further validate the identification of target compounds. This combined approach ensures the reliability and reproducibility of our GC-MS analysis.

### 2.3 Cell culture and cell viability analysis

HepG2 cells were purchased from the Chinese Academy of Sciences (Shanghai, China) and cultured in DMEM medium containing 10% FBS and 1% penicillin/streptomycin, at 37°C with 5% CO_2_. HepG2 cells (1 × 10^4^ per well) were seeded into a 96-well plate. Post-adhesion, the cells were treated with various concentrations of oleic acid (0, 0.1, 0.2, 0.4, 0.6, 0.8, 1.0%), citrus reticulata volatile oil (0, 30, 60, 120, 240, 480 mg/L), atractylon (0, 30, 60, 120, 240, 480 mg/L), D-limonene (0, 30, 60, 120, 240, 480 mg/L), and γ-elemene (0, 30, 60, 120, 240, 480 mg/L) in complete culture medium for 24 h. After 24 h, 100 μL of a 10% CCK-8 solution was added to each well, and the absorbance values were measured after 1 h to calculate the cell survival rate.

### 2.4 Lipid content analysis

For lipid content analysis, HepG2 cells (2 × 10^5^ per well) were seeded in a 6-well plate and incubated with medium containing 0.6% oleic acid for 24 h according to the method described in the literatures (Fu et al., 2022; Yuri et al., 2023). At the completion of incubation, the medium was exchanged for new culture medium with or without 240 mg/L ZBEO, 240 mg/L atractylon, 240 mg/L D-limonene, and 240 mg/L γ-elemene. After additional 24 h, cells were stained according to the protocol provided with the Oil Red O staining kit (Solaibao, Beijing, China) and subsequently visualized under an inverted microscope. Following this, 200 μL of isopropanol was used to extract the cellular dye. An aliquot of 100 μL mixture was then transferred to a 96-well plate, and the absorbance was measured at 506 nm to determine the intracellular lipid content.

To determine the intracellular TC and TG levels, cells were seeded into a 96-well plate and subjected to the same drug concentration treatment. Following treatment, the cells were rinsed twice with PBS, and lysed using RIPA buffer (Solarbio, Beijing, China). After centrifugation, the supernatant was collected and used for the quantification of TC and TG according to the manufacturer’s instructions (Jiancheng Bioengineering, Nanjing, China).

### 2.5 Animals and experimental design

Ninety male SD rats (SPF, 6 weeks old, weight 170–210 g) were purchased from Jiangxi University of Chinese Medicine (JXUTCM) (SCXK (Gan) 2018-0033). All the experiments were approved by the Experimental Animal Science and Technology Center of JXUTCM (Jiangxi, China, Certificate No: SYXK (Gan) 2022-0002). All procedures were performed in compliance with the requirements of experimental protocols approved by the Ethics Committee for Animal Experiments of JXUTCM (JZLLSC20220797). Dose selection for ZBEO was based on traditional prescriptions and clinical human doses, translated to animal doses using body surface area scaling. The initial dose was set at approximately 5%, with 2.5% and 10% doses selected to explore the dose-response relationship.

After 1 week of adaptation period, all rats were randomly divided into normal control group and high-fat diet (HFD) group. The rats in the control group received a standard chow diet supplying 10% of energy as fat and those in the HFD supplying 24% of energy as fat. The obese rat model was successfully established when the body weight of HFD group was significantly higher than that of normal control group (*P* < 0.05). Subsequently, the obese rats were randomly allocated into five groups which continued to receive the HFD: obese rat model group (model group); obese rats received topical application of 1 mL of 2.5% ZBEO on the abdominal skin and given an equal amount of saline (L-ZBEO); obese rats received topical application of 1 mL of 5.00% ZBEO on the abdominal skin and given an equal amount of saline (M-ZBEO); and obese rats received topical application of 1 mL of 10.00% ZBEO on the abdominal skin and given an equal amount of saline (H-ZBEO); obese rats received intragastric administration of 2 mL/500 g of orlistat with a concentration of 9.41 mg/mL and topical application 1 mL *Simmondsia chinensis* EO on the abdominal skin as positive group (P group). The rats in the normal control group were given an equal amount of saline and topical application 1 mL *S. chinensis* EO on the abdominal skin; eight rats in each group. During the 5-week experiment, the food intake and body weights of rats were measured weekly. Two hours after the last treatment, rat feces were collected using the stress method and stored at −80°C for microbiome analysis. After a 12 h fasting period, blood samples were taken from the abdominal aorta and centrifuged to obtain serum. Finally, the hypothalamus, colon, liver, white adipose and brown adipose tissues were extracted. The flowchart of the animal experiment is shown in Supplementary Figure S1.

### 2.6 Biochemical analysis

The levels of TC, TG, HDL-c, LDL-c, apolipoprotein A1 (Apo A1), bile acids (BAs), creatinine, urea, glucose, and aspartate aminotransferase (AST)/alanine transaminase (ALT) in serum were measured using an automated biochemical analyzer (P800, Roche modular, Germany).

### 2.7 Detection of inflammatory factors and adipocytokines

The contents of IL-6, IL-17, TNF-α, visfatin, adiponectin, APQ7, GLP-1, leptin, orexin A, CCK, and ghrelin in serum were determined by ELISA kits according to the manufacturer’s instructions.

### 2.8 Pathological evaluation

White adipose, brown adipose, and liver tissues were fixed with 4% paraformaldehyde for 10 min and then frozen sections were stained using H&E or Oil Red O staining according to standard experimental procedures. Subsequently, the stained slices were observed using an optical microscope (Leica Microsystems GmbH, MD1000) with a ×100 objective lens.

### 2.9 Quantitative real-time PCR (RT-qPCR)

Total RNA was isolated from rat hypothalamic tissue using TRIzol, and cDNA was synthesized by reverse transcription and amplified. Sequences of primers are presented in Table 1. The transcription levels of each target gene (AMPK-α, POMC, NPY) were normalized to GAPDH and expressed as fold changes relative to the indicated control using the 2^−ΔΔCT^ method to determine the relative expression levels of each gene.

**TABLE 1:** Sequence information of *AMPK-α*, *POMC*, and *NPY* primers.

Target gene	Primer sequence 5′-3′		GenBank number
*GAPDH*	Forward	GAC​CAC​AGT​CCA​TGC​CAT​CA	NM_017008.4
	Reverse	CAC​AGG​AGA​CAA​CCT​GGT​CC	
*NPY*	Forward	GTG​TTT​GGG​CAT​TCT​GGC​TG	NM_012614.2
	Reverse	TTC​AAG​CCT​TGT​TCT​GGG​GG	
*AMPK-α*	Forward	CGA​GCT​ATG​AAG​CAG​CTG​GA	NM_023991.1
	Reverse	AGC​GCT​GAG​GTG​TTG​AAG​AA	
*POMC*	Forward	CAA​CGG​AGA​TGA​ACA​GCC​CT	NM_139326.3
	Reverse	GCG​ACA​TTG​GGG​TAC​ACC​TT	

### 2.10 Western blotting

Hypothalamus tissues were shredded and homogenized in RIPA buffer, and then centrifuged to obtain protein. The protein content was quantified with BCA protein assay kit. Proteins were mixed with loading buffer and denatured by being boiled at 97°C for 6 min. Equal content of proteins (30 µg) was separated by 8%–12% SDS-PAGE gels and subsequently transferred onto 0.22 μm PVDF membranes. Afterwards, the membrane was blocked with 5% BSA solution at room temperature for 1.5 h, and then incubated with primary antibodies, mouse Anti-POMC (4752), rabbit Anti-AMPK-α (5831), rabbit Anti-CART (14,437) and rabbit Anti-NPY (11,976) (purchased from Sanying Biotechnology, Wuhan, China), and β-Actin (1:1000) overnight at 4°C. Then, the membranes were incubated with secondary antibodies goat anti-rabbit IgG (H + L), HRP conjugated (1:3000) at room temperature for 1 h. After incubating with a chemiluminescence ECL assay reagents, the immunoreactive bands were visualized using the ChemiScope Mini 3300 Chemiluminescence Imaging. The band densities were quantified by ImageJ software.

### 2.11 Analysis of UPLC-Q-TOF-MS

100 μL of serum sample was mixed with 300 μL of methanol. The mixture was vortexed for 60 s, followed by centrifugation at 4°C and 12,000 rpm for 15 min. The supernatant was collected and freeze-dried. Simultaneously, 50 μL of 70% methanol was added to the residue, and the mixture was centrifuged again at 4°C and 12,000 rpm for 15 min. The supernatant was then collected for further analysis. UHPLC-Q-TOF-MS was employed to analyze the serum samples in positive ion mode. The Vanquish UHPLC column (Thermo Fisher Scientific, USA) (100 mm × 2.1 mm, 1.8 μm) was used with a mobile phase of 0.1% formic acid aqueous solution (A) and acetonitrile (B). The gradient elution was as follows: 0–11 min, 85%–25% A; 11–12 min, 25%–2% A; 12–14 min, 2%–2% A; 14–14.1 min, 2%–85% A; 14.1–16 min, 85% A. The column temperature was set at 30°C, and the flow rate was 0.1 mL/min. A volume of 2 μL was injected. ESI electrospray ionization source was used with a scanning range of m/z 100–1500, spray voltage of ±3.4 kV, and ion source temperature of 500°C. The data was imported into Progenesis QI software for denoising, peak extraction, peak identification, and normalization based on total peak area. Then, the data was imported into SIMCA (V16.0.2) software for principal component analysis (PCA) and orthogonal partial least squares-discriminant analysis (OPLS-DA). Differential metabolites were selected based on variable important in projection (VIP) > 1.0 and *P* < 0.05, and identified using the HMDB database. The differential metabolites were then imported into Metaboanalyst 5.0 online platform for metabolic pathway enrichment analysis.

### 2.12 16S rRNA gene-based bacterial community analysis

The gut microbial community of rats was examined through 16S rRNA gene sequencing. Genomic DNA was extracted from rat feces using a dedicated extraction kit. The quality and concentration of the resulting DNA were assessed via 1% agarose gel electrophoresis and spectrophotometry. Paired-end sequencing was conducted on the Illumina MiSeq PE300 platform, utilizing primers 515F (5-GTGCCAGCMGCCGCGGTAA-3) and 806R (5-GGACTACHVGGGTWTCTAAT-3) to amplify the V4 region of the bacterial 16S rRNA gene. The PCR products were validated through gel electrophoresis to ensure proper size, followed by purification with the Agencourt AMPure XP kit. The raw sequence data was deposited in the NCBI SRA database. Reads were paired and optimized using the RDP classifier for OTU clustering at 97% similarity. Diversity analyses, along with Linear Discriminant Analysis Effect Size (LEfSe), were carried out on all samples. Additionally, Spearman’s correlation analysis was employed to explore relationships with environmental factors and metabolic differences.

### 2.13 Statistical analysis

Data normality and homogeneity of variances were confirmed using the Shapiro-Wilk and Levene’s tests, respectively. One-way ANOVA was performed to compare the means among multiple groups. To identify the specific differences between groups while controlling for Type I errors, we conducted Tukey’s *post hoc* test. All statistical analyses were performed using GraphPad Prism 8.

## 3 Results

### 3.1 Identification and *in vitro* biological activity analysis of ZBEO components

**FIGURE 1:**
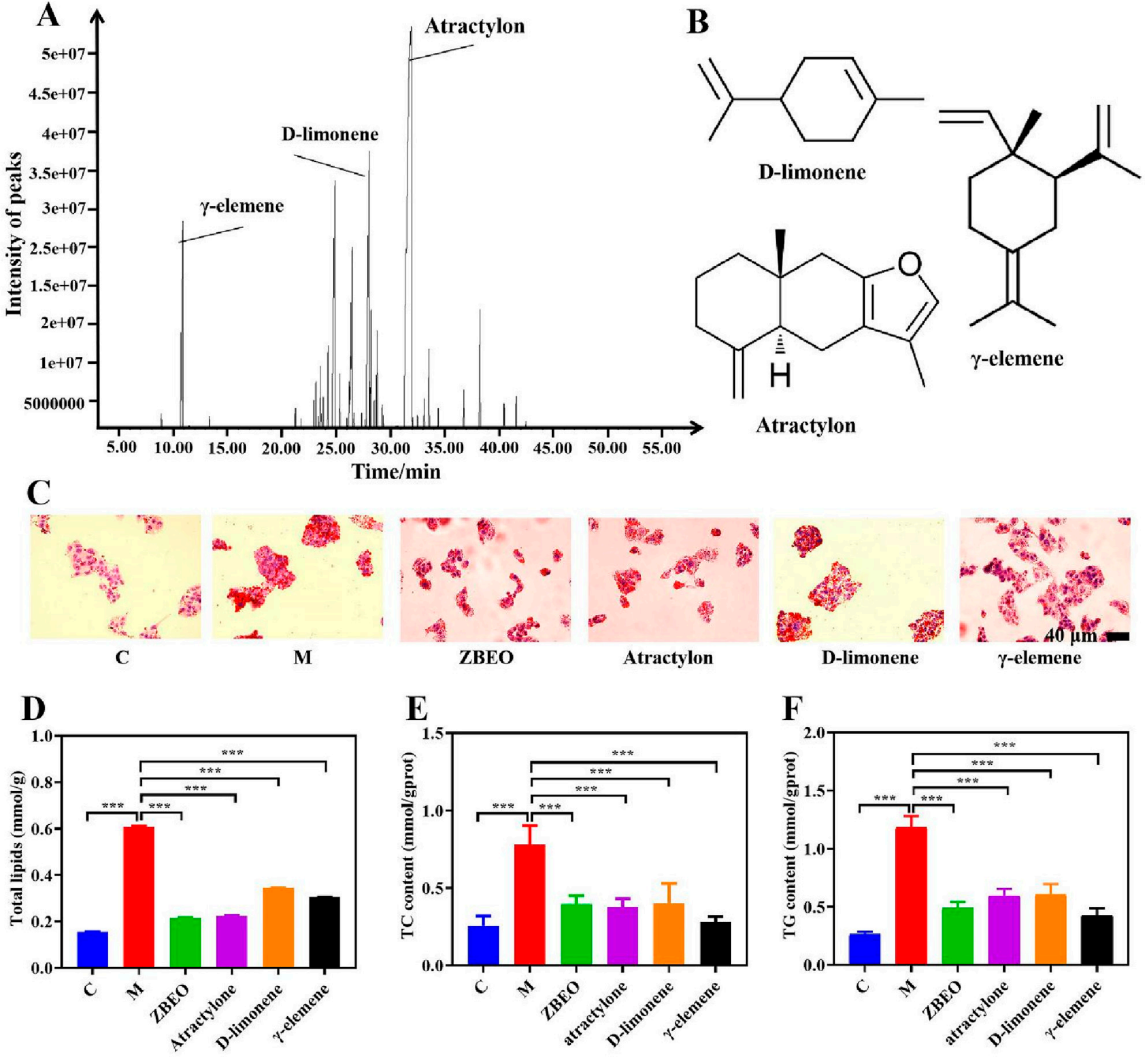
The composition and biological activity assessment of ZBEO. **(A)** GC-MS profile of ZBEO; **(B)** Structures of D-limonene, atractylon, and γ-elemene. **(C)** Lipid deposition in HepG2 cells analyzed by Oil Red O staining (scale bar = 40 μm). **(D)** The content of total lipid. **(E)** The content of TC. **(F)** The content of TG. Statistical analysis was performed using ANOVA and Student’s t test. ***P < 0.001 represented statistical significance. Data were expressed as the mean ± SD, n = 6. C: normal control group; M: model group, ZBEO: *Citrus aurantium L.-Atractylodes macrocephala Koidz* essential oil.

The composition of ZBEO was analyzed by GC-MS. Based on the NIST library matching results and the percentage of retention time and peak area of the spectral peaks, the results revealed that ZBEO was composed of 46 components (Supplementary Table S1), among them, atractylon, D-limonene and γ-elemene were the main components, accounting for 40.82%, 8.98% and 7.02%, respectively (Figure 1A). The structures of these components were shown in Figure 1B. As the total contents of atractylon, D-limonene and γ-elemene exceeded 50% of ZBEO, they might play crucial roles in the active function of ZBEO.

CCK-8 assay was used to measure the viability of HepG2 cells, which is commonly utilized for *in vitro* analysis of lipid accumulation (Fu et al., 2022; Yuri et al., 2023). To ensure that the drug treatments exerted no significant inhibitory effects on cell proliferation, 0.6% oleic acid, 240 mg/L ZBEO, 240 mg/L atractylon, 240 mg/L D-limonene, and 240 mg/L γ-elemene were selected for experiments (Supplementary Figure S2). Oil Red O staining revealed that the oleic acid-treated cells exhibited round, enlarged morphology with the presence of lipid droplets, while this phenomenon could be effectively attenuated by treatment with ZBEO or its main chemical composition (Figure 1C). Further investigation revealed that treatment with ZBEO or its main components significantly reduced the levels of total lipids, as well as intracellular TG and TC, compared to oleic acid alone (*P* < 0.001) (Figures 1D, F). Interestingly, the inhibitory effect of atractylon on lipid accumulation was comparable to that of ZBEO and better than that of D-limonene and γ-elemene at equivalent dose (Figure 1D). These results indicated that ZBEO could reverse lipid accumulation in liver cells, which might be attributed to atractylon.

### 3.2 ZBEO significantly alleviates body weight and ameliorates dysregulation of glucose-lipid metabolism in HFD-induced rats

**FIGURE 2:**
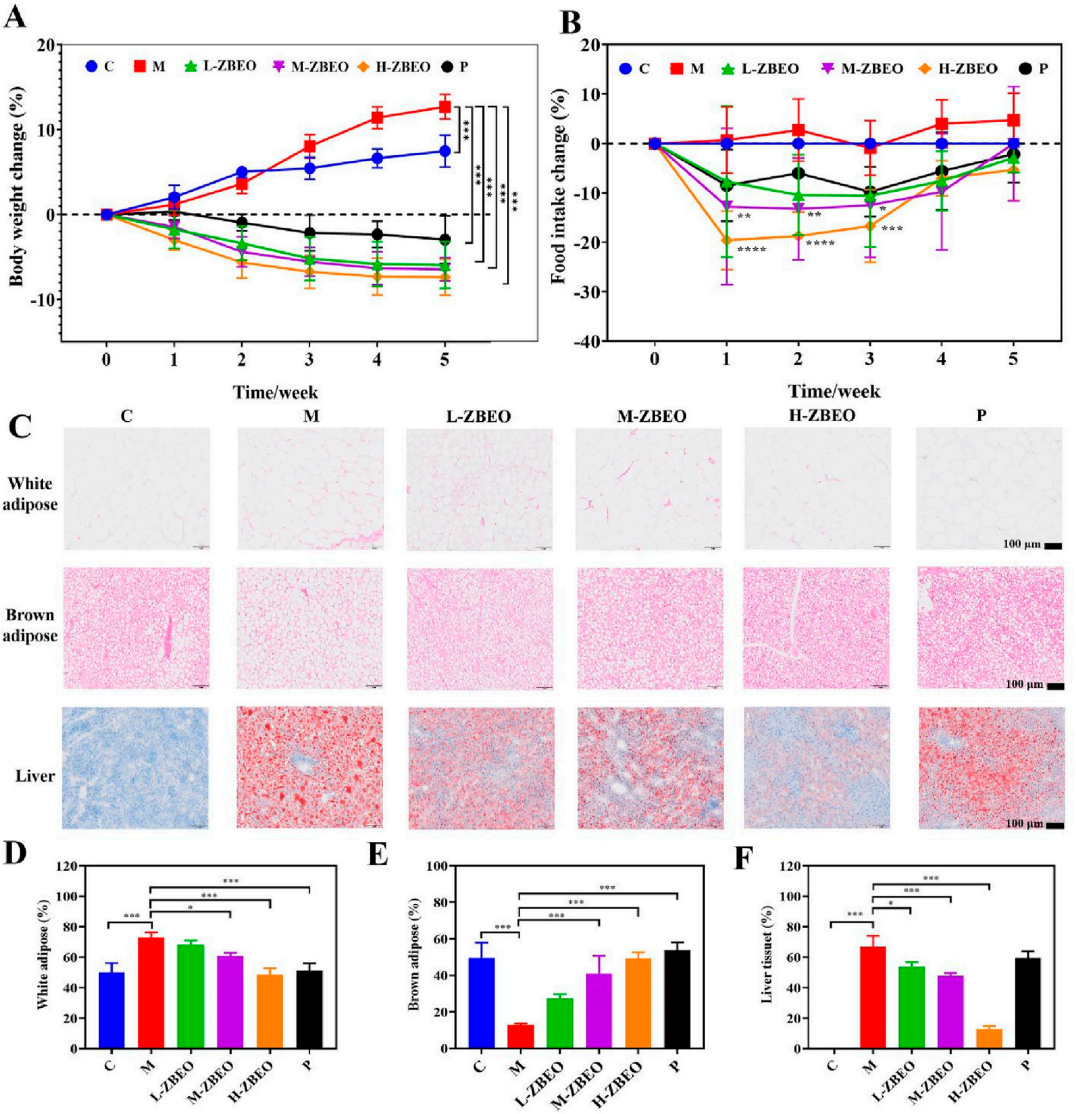
Effects of ZBEO on body weight and glucose and lipid metabolism in HFD-fed rats. **(A)** Body weights change (n = 8). **(B)** Food intake change (n = 8). **(C)** Histopathological analysis of metabolic tissues in HFD-induced obese rats. Top panel: Representative H&E-stained sections of white adipose tissue and brown adipose tissue (Scale bar = 100μm). Bottom panel: Oil Red O-stained liver sections (Scale bar = 100 μm). **(D)** Percentage of white adipose tissue adipocytes >80 microns in diameter (n = 3). **(E)** Percentage of brown adipose tissue adipocytes >20 microns in diameter (n = 3). **(F)** Percentage of area of liver tissue fat (n = 3). Statistical analysis was performed using ANOVA and Student’s t test. *P < 0.05, **P < 0.01, and ***P < 0.001 represented different statistical significances. Data were expressed as the mean ± SD. ZBEO: *Citrus aurantium L.-Atractylodes macrocephala Koidz* essential oil; C: normal control group; M: model group; P: orlistat group; L-ZBEO: 2.50% ZBEO dosage group; M-ZBEO: 5.00% ZBEO dosage group; H-ZBEO: 10.00% ZBEO dosage group.

The *in vivo* effect of ZBEO on obesity was evaluated using HFD-obese rat model. Following a 5-week treatment regimen with 1 mL jojoba oil or 1 mL jojoba oil supplemented with 2.5% ZBEO, 5% ZBEO or 10% ZBEO, a significant increase in body weight was observed in the model group when compared to the normal control group (*P* < 0.001) (Figure 2A). In contrast, the groups treated with ZBEO exhibited a significant reduction in body weight (*P* < 0.001) (Figure 2A). Moreover, there was no significant difference between ZBEO groups and orlistat group. During weeks 1–3, the model group exhibited greater food intake than the normal control group, whereas the ZBEO groups had reduced intake compared to the model group (Figure 2B). Notably, in weeks 3–5, the ZBEO-treated group showed a gradual increase in food intake, which was consistent with the trend of slowing down of the rate of weight loss in weeks 3–5, and we speculated by that it might have entered a plateau period of weight loss. Furthermore, the model group displayed massive lipid accumulation in white adipose, and liver tissues, and a significant decrease in brown adipose. (Figure 2C). However, intervention with different doses of ZBEO or orlistat visibly inhibited this phenomenon, and the effect of ZBEO showed a dose-dependent trend (Figures 2C–F). These findings suggest that ZBEO possesses an obvious effect on reducing body weight in HFD-obese rat, which may be related to appetite regulation.

Obesity is usually accompanied by the disorder of glucose-lipid metabolism, and impaired liver and kidney function. Therefore, we further evaluated the related regulatory effect of ZBEO in the obese rats (Table 2). The results revealed that, compared with the normal control group, the model group exhibited significantly decreased levels of HDL-c (*P* < 0.001) and Apo A1 (*P* < 0.01), while the levels of LDL-c, TG, TC, urea, glucose, and bile acid were significantly increased (*P* < 0.001) (Table 2). However, treatment with ZBEO or orlistat effectively reversed this phenomenon. Specifically, H-ZBEO significantly reduced the levels of LDL-c, TG, TC, urea, glucose, and bile acids (*P* < 0.001), and significantly increased the levels of HDL-c and Apo A1 (*P* < 0.01) (Table 2). This suggests that ZBEO, particularly at higher doses, may have a beneficial effect on lipid and glucose metabolism, similar to the effects observed with orlistat treatment. In addition, the ratio of ALT/AST is related to liver function. When the ratio of ALT/AST is elevated in obese patients, it suggests the possibility of metabolic syndrome, extensive liver damage, and poor prognosis (Kim et al., 2006; Carrillo-Iregui et al., 2010; Uemura et al., 2014). Creatinine can reflect the glomerular filtration function of the kidneys. An increase in creatinine indicates kidney damage and can also be used as one of the indicators for evaluating hypertension (Kim et al., 2006; Carrillo-Iregui et al., 2010). Compared with the control group, the ALT/AST and creatinine levels of the model group were significantly increased, and after ZBEO treatment, H-ZBEO was able to significantly reduce the level of ALT/AST among serum (*P* < 0.05). In addition, the intervention effect on serum creatinine level was not significant in each dose group of ZBEO (Table 2). These results indicated that ZBEO could maintain normal glucose and lipid metabolism as well as repair liver functions in HFD-fed rats.

**TABLE 2:** Serum biomarkers of glucose and lipid metabolism in each group (Means ± SD, n = 8).

Group Marker	C	M	P	L-ZBEO	M-ZBEO	H-ZBEO
AST/ALT	3.13 ± 0.56	4.37 ± 0.37 ^###^	3.16 ± 0.72 ^***^	4.23 ± 0.36	3.82 ± 0.26	3.56 ± 0.63 ^*^
LDL-c	1.13 ± 0.10	2.60 ± 0.32 ^###^	1.73 ± 0.16 ^***^	2.33 ± 0.32	1.95 ± 0.30 ^***^	1.85 ± 0.25 ^***^
HDL-c	0.79 ± 0.08	0.44 ± 0.08 ^###^	0.59 ± 0.10 ^**^	0.53 ± 0.03	0.56 ± 0.05 *	0.58 ± 0.08 ^**^
Apo A1	0.27 ± 0.01	0.22 ± 0.02 ^##^	0.25 ± 0.03	0.23 ± 0.03	0.25 ± 0.01	0.26 ± 0.02 ^**^
TG	0.68 ± 0.08	0.96 ± 0.14 ^###^	0.76 ± 0.13 ^**^	0.91 ± 0.09	0.77 ± 0.08 ^**^	0.64 ± 0.07 ^***^
TC	2.10 ± 0.19	3.56 ± 0.35 ^###^	2.64 ± 0.21 ^***^	2.96 ± 0.48 ^**^	2.91 ± 0.0.39 ^**^	2.65 ± 0.21 ^***^
Urea	5.02 ± 0.22	7.15 ± 0.82 ^###^	5.99 ± 0.43 ^**^	6.41 ± 0.69	6.23 ± 0.51 ^*^	5.31 ± 0.59 ^***^
Creatinine	78.60 ± 5.65	87.76 ± 3.56 ^##^	83.03 ± 3.23	92.50 ± 7.45	81.43 ± 4.57	80.63 ± 5.00
Glucose	9.44 ± 1.55	12.58 ± 1.06 ^###^	10.08 ± 1.44 ^*^	10.65 ± 1.42	10.34 ± 1.09 ^*^	9.20 ± 1.03 ^***^
Bile acids	74.14 ± 22.80	130.10 ± 20.75 ^###^	98.11 ± 10.19 ^*^	73.16 ± 14.21 ^***^	71.08 ± 18.32 ^***^	67.76 ± 17.32 ^***^

Note: ZBEO: *Citrus aurantium L.-Atractylodes macrocephala Koidz* essential oil; C: normal control group; M: model group; P: orlistat group; L-ZBEO: 2.50% ZBEO, dosage group; M-ZBEO: 5.00% ZBEO, dosage group; H-ZBEO: 10.00% ZBEO, dosage group. Statistical analysis was performed using ANOVA, and Student’s t-test. ^#^ indicates comparison with C group, ^##^ *P* < 0.01, ^###^ *P* < 0.001; * indicates comparison with M group, * *P* < 0.05, ** *P* < 0.01, and *** *P* < 0.001 represented different statistical significances. Data were expressed as the mean ± SD, n = 8.

### 3.3 ZBEO suppresses HFD-induced increase in inflammatory factors and change in adipocytokines levels

**FIGURE 3:**
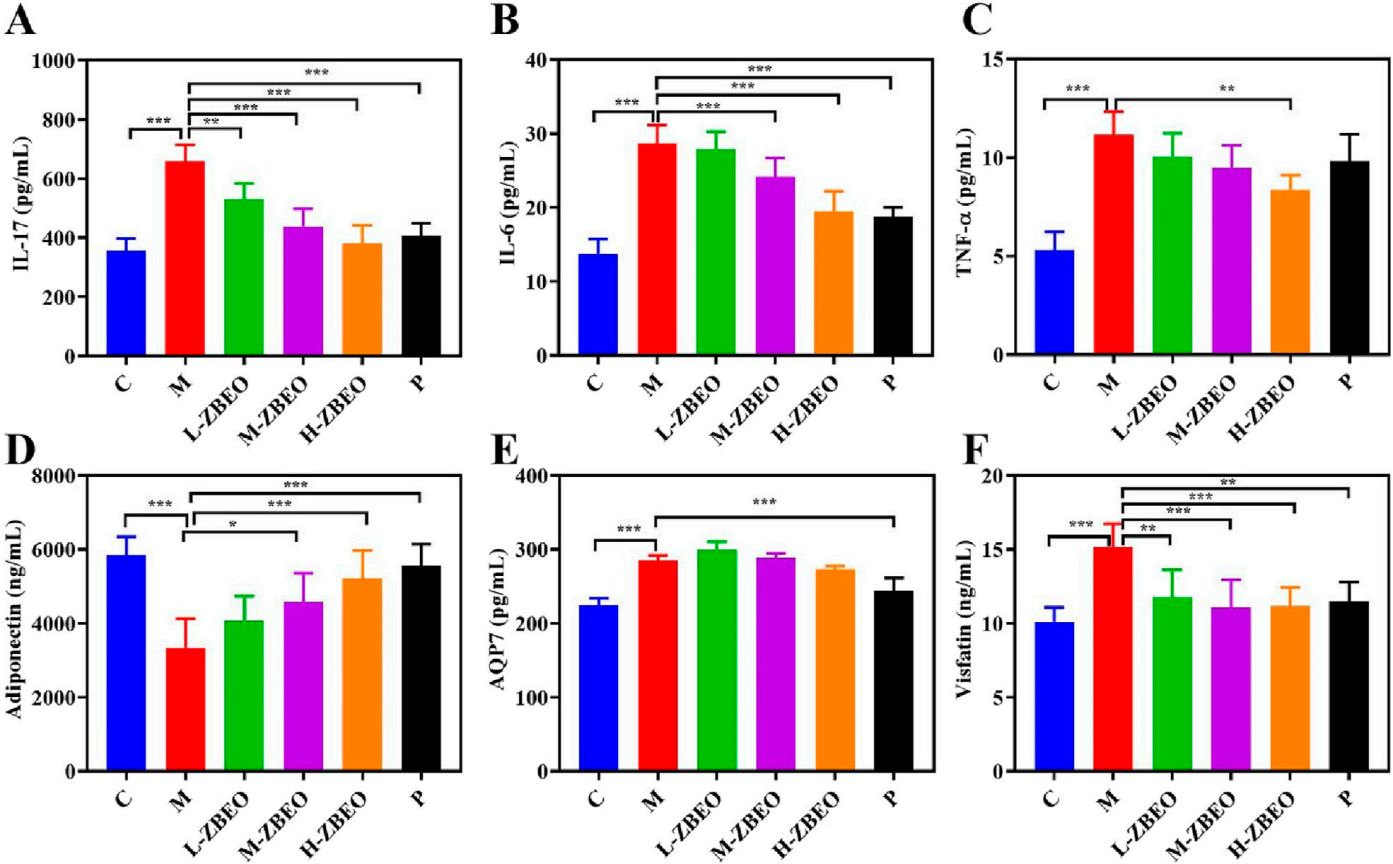
Effects of ZBEO on the levels of serum inflammatory factors and adipocytokines in HFD-induced rats. **(A)** IL-17. **(B)** IL-6. **(C)** TNF-ɑ. **(D)** Adiponectin. **(E)** AQP7. **(F)** Visfatin. Statistical analysis was performed using ANOVA and Student’s t test. *P < 0.05, **P < 0.01, and ***P < 0.001 represented different statistical significances. Data were expressed as the mean ± SD, n = 8. ZBEO: *Citrus aurantium L.-Atractylodes macrocephala Koidz* essential oil; C: normal control group; M: model group; P: orlistat group; L-ZBEO: 2.50% ZBEO dosage group; M-ZBEO: 5.00% ZBEO dosage group; H-ZBEO: 10.00% ZBEO dosage group.

Obesity is associated with an increase in systemic inflammatory markers, which exacerbates the risk of developing complications (Terzo et al., 2023). To determine whether ZBEO could ameliorate systemic inflammation of obese rats, the levels of pro-inflammatory cytokines IL-17, IL-6, and TNF-α were measured. The model group markedly showed higher serum IL-17, IL-6, and TNF-α levels than the normal control group (*P* < 0.001) (Figures 3A–C). However, ZBEO treatment inhibited the excess secretion of IL-6, IL-17, and TNF-α in a dose-dependent manner (Figures 3A–C). Additionally, ZBEO inhibited the changes in serum adipokine levels induced by an HFD. Compared to the model group, both M-ZBEO and H-ZBEO effectively increased the content of adiponectin (Figure 3D), which is related to insulin resistance and elevated glucose levels. Interestingly, orlistat significantly reduced the levels of AQP7, which plays a crucial role in glycerol transport, while the effect of ZBEO was not pronounced (Figure 3E). Moreover, all doses of ZBEO significantly decreased the content of visfatin in the serum (Figure 3F), suggesting that ZBEO possesses anti-inflammatory and improved insulin resistance effects. These results suggested that the anti-obesity effect of ZBEO may partly be attributed to its ability to regulate anti-inflammatory factors and adipocytokines.

### 3.4 ZBEO regulates appetite-related gastrointestinal hormone secretion through POMC/CART signaling pathway in the obese rats

**FIGURE 4:**
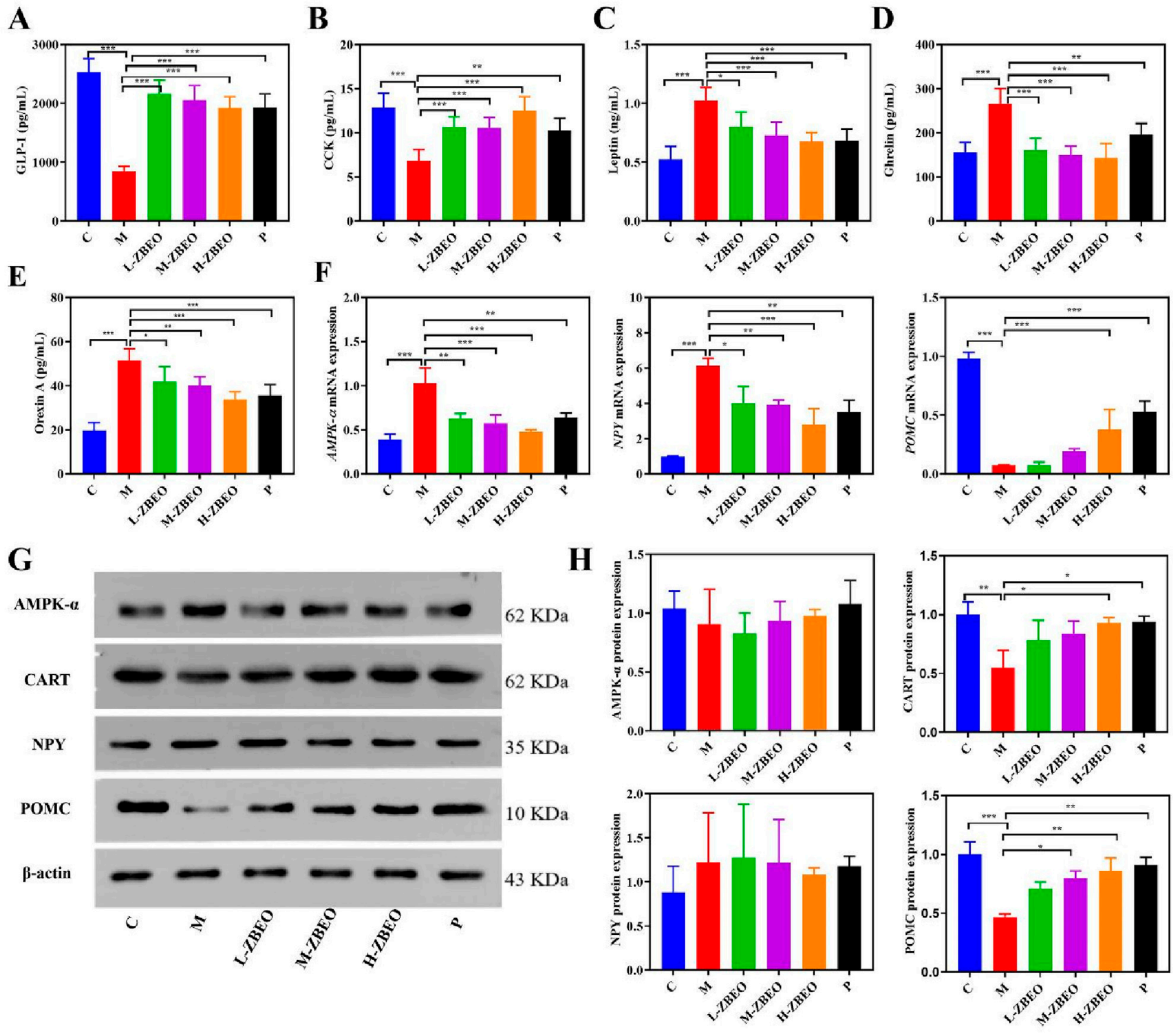
Effects of ZBEO on appetite-related gastrointestinal hormones in HFD-fed rats. **(A)** The content of GLP-1 in serum (n = 6). **(B)** The content of CCK in serum (n = 6). **(C)** The content of leptin in serum (n = 6). **(D)** The content of ghrelin in serum (n = 6). **(E)** The content of orexin A in serum (n = 6). **(F)** The mRNA level of *AMPK-α, POMC* and *NPY* in the hypothalamus (n = 5). **(G)** The level of AMPK-α, POMC, NPY, CART proteins, and β-actin content was used as the loading control in the hypothalamus (n = 3). **(H)** Quantification of AMPK-α, POMC, NPY and CART protein expression levels in the hypothalamus **(G)** (n = 3). Statistical analysis was performed using ANOVA and Student’s t test. *P < 0.05, **P < 0.01, and ***P < 0.001 represented different statistical significance. Data were expressed as the mean ± SD. ZBEO: *Citrus aurantium L.-Atractylodes macrocephala Koidz* essential oil; C: normal control group; M: model group; P: orlistat group; L-ZBEO: 2.50% ZBEO dosage group; M-ZBEO: 5.00% ZBEO dosage group; H-ZBEO: 10.00% ZBEO dosage group.

The underlying mechanism by which ZBEO achieved treatment against obesity was explored. To elucidate the influence of ZBEO on appetite regulation, we examined serum levels of key gastrointestinal hormones associated with satiety and hunger. Specifically, the concentrations of orexin A, ghrelin, leptin, CCK, and GLP-1 were measured in HFD-induced obese rats following ZBEO treatment. Relative to those in the normal control group, the rats in the model group showed decreases in CCK and GLP-1 levels, and increases in leptin, ghrelin and orexin A levels, indicating that obesity could result in the imbalance in appetite-related gastrointestinal hormones (Figures 4A–E). However, ZBEO treatment effectively reversed this effect with comparable outcome with orlistat (Figures 4A–E), suggesting that ZBEO exhibits distinct regulation on appetite suppression.

The hypothalamus is the main site of action for appetite-regulating hormones. Two important neuropeptides, POMC and NPY, are involved in feeding and energy homeostasis and serve as key hypothalamic targets for appetite-regulating hormones. In addition, appetite-regulating hormones have been shown to exert an appetite-suppressing effect by decreasing NPY secretion and increasing CART expression through an AMPK-dependent mechanism. Therefore, we investigated the mechanisms by which ZBEO regulates appetite-related gastrointestinal hormones through exploring the NPY/AMPK/POMC/CART signaling pathway. First, RT-qPCR analysis revealed that ZBEO decreased the mRNA levels of NPY and AMPK-α while increasing the mRNA level of POMC (Figure 4F). Compared with the model group, ZBEO significantly enhanced the protein expression of CART and POMC, but had no significant effect on the protein expression of AMPK-α and NPY (Figures 4G, H). These findings indicated that ZBEO may exert its effects on improving feeding mechanisms in obese rats by potentially influencing levels of appetite-related gastrointestinal hormones and modulating the POMC/CART signaling pathway.

### 3.5 Effect of ZBEO on serum metabolome

**FIGURE 5:**
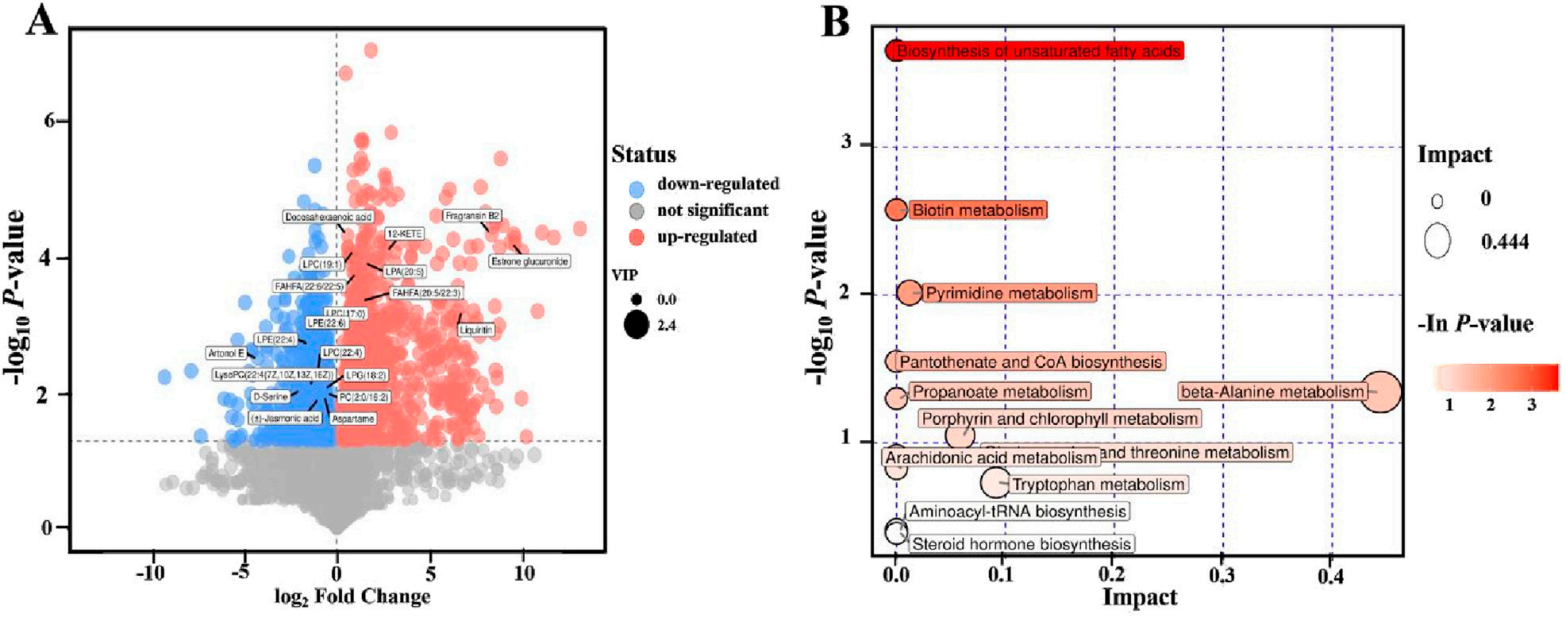
Effects of ZBEO on serum metabolite profiles. **(A)** Volcanic map of differential metabolites (M vs. H-ZBEO) (OPLS-DA). **(B)** Bubble map of KEGG pathway analysis. ZBEO: *Citrus aurantium L.-Atractylodes macrocephala Koidz* essential oil; C: normal control group; M: model group; H-ZBEO: 10.00% ZBEO dosage group.

An untargeted metabolomic analysis was conducted to explore the impact of ZBEO treatment on serum metabolites. The PCA results revealed that the model group and normal control group were completely separated from each other, and that the serum metabolic pattern of H-ZBEO group was significantly different from that of the model group, indicating ZBEO could alter the metabolism of HFD rats (Supplementary Figure S3). The volcanic map in Figure 5A presented the differential metabolites. A total of 20 differential metabolites were identified between model group and H-ZBEO group, and possible metabolic pathways affected by ZBEO were identified by the pathway enrichment analysis (Figure 5B). Additionally, pathway enrichment analysis was performed on the differentially metabolites identified in Figure 5A, with a screening condition of VIP ≥1. It was found that ZBEO primarily alleviated obesity through the modulation of 12 metabolic pathways, including the biosynthesis of unsaturated fatty acids, biotin metabolism, and pyrimidine metabolism (Figure 5B). These results indicate that the metabolic phenotype of the obese rats undergoes changes, and ZBEO treatment might improve the host metabolism of obese individuals by regulating obesity-related chronic inflammation, energy metabolism, and glucose-lipid metabolism.

### 3.6 ZBEO attenuates gut microbiota dysbiosis in obese rats

**FIGURE 6:**
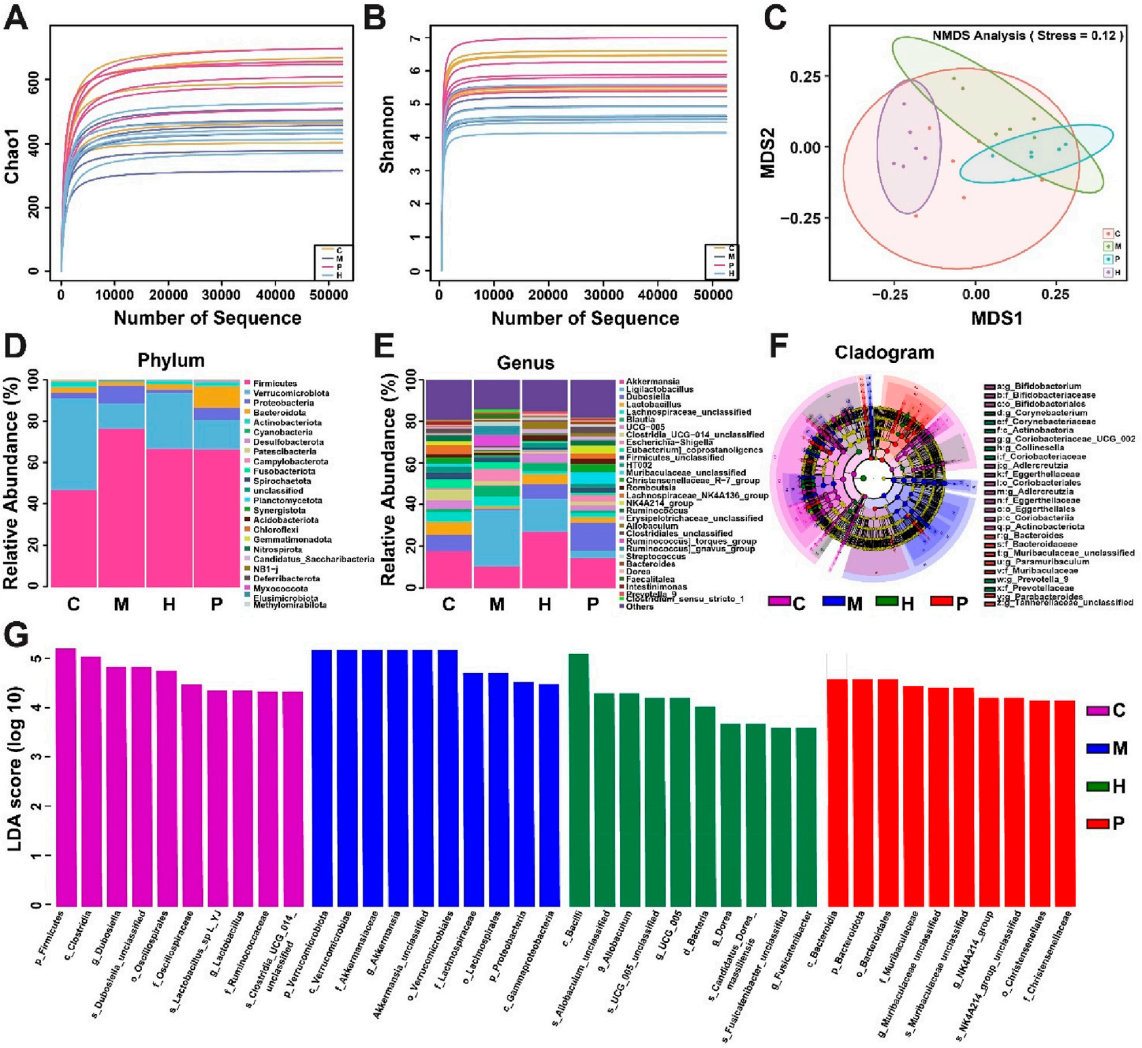
Effects of ZBEO on the gut flora in obese rats. **(A)** α-diversity analyses of intestinal flora measured by chao1 index. **(B)** α-diversity analyses of intestinal flora measured by Shannon index. **(C)** β-diversity analyses of the composition of bacterial communities. **(D, E)** Abundance of intestinal flora at the phylum and genus levels; **(F)** Evolutionary branch diagram. **(G)** LDA value distribution histogram, with taxa meeting LDA score threshold >4 being listed. ZBEO: *Citrus aurantium L.-Atractylodes macrocephala Koidz* essential oil; C: normal control group; M: model group; P: orlistat group; H: 10.00% ZBEO dosage group.

Previous studies indicated that dysregulation of the gut microbiota may lead to the development of obesity (Paturi et al., 2017; Zou et al., 2018). Therefore, we studied the composition of intestinal flora using 16S rRNA gene sequencing to explore the regulatory effect of ZBEO on gut microbiota. α and β diversities are mainly used as indicators to evaluate the microbial diversity. According to α and β diversities analysis, the gut microbial diversity in model group was lower than that of normal control group, while this effect could be reversed by ZBEO intervention (Figures 6A–C).

The gut microbiota at the phylum and genus levels was detected to further analyze the differences in the relative abundance of gut microbiota in each group. Analysis at the phylum level revealed that the major microbial taxa were *Firmicutes*, *Verrucomicrobiota*, *Proteobacteria*, *Bacteroidota*, and *Actinobacteriota*, with *Cyanobacteriota* being the most dominant phylum (Figure 6D). It should be noted that the relative abundance of *Firmicutes* and *Proteobacteria* in the model group was significantly higher than that in the normal control group, while that of *Verrucomicrobiota* was significantly lower (Figure 6D). However, H-ZBEO treatment reduced the abundance of *Firmicutes* and *Proteobacteria*, and elevated that of *Verrucomicrobiota* in obese rats (Figure 6D). The gut microbiota at the genus level indicated that the model group had a distinct microbial profile relative to the normal control group. HFD mainly induced an increase in *Ligilactobacillus*, and decreases in *Akkermansia*, *Dubosiella* and *Lactobacillus*. In contrast, the results of H-ZBEO treatment for 5 weeks were opposite to the model group (Figure 6E).

To identify bacterial taxa that differed significantly between groups, LEfSe (values >3) analysis was performed between groups (Figures 6F, G). From phylum to genus, a total of 85 taxa were obtained from all groups. Among them, the normal control group was enriched to 65 taxa, the model group to 44 taxa, the P group to 40 taxa, the H-ZBEO group to 35 taxa. The results showed that p_*Firmicutes*, c_*Clostridia* and g_*Dubosiella* were more abundant in the normal control group; p_*Verrucomicrobiota*, c_*Verrucomicrobiae* and f_*Akkermansiaceae* were more abundant in the model group; c_*Bacteroidia*, p*_Bacteroidota* and o_*Bacteroidale* were more abundant in the P group. In the H-ZBEO group, the abundance of c_*Bacilli*, s_*Allobaculum unclassified* and g_*Allobaculum* were more abundant.

### 3.7 Correlation analysis between gut microbiota and obese biomarkers

**FIGURE 7:**
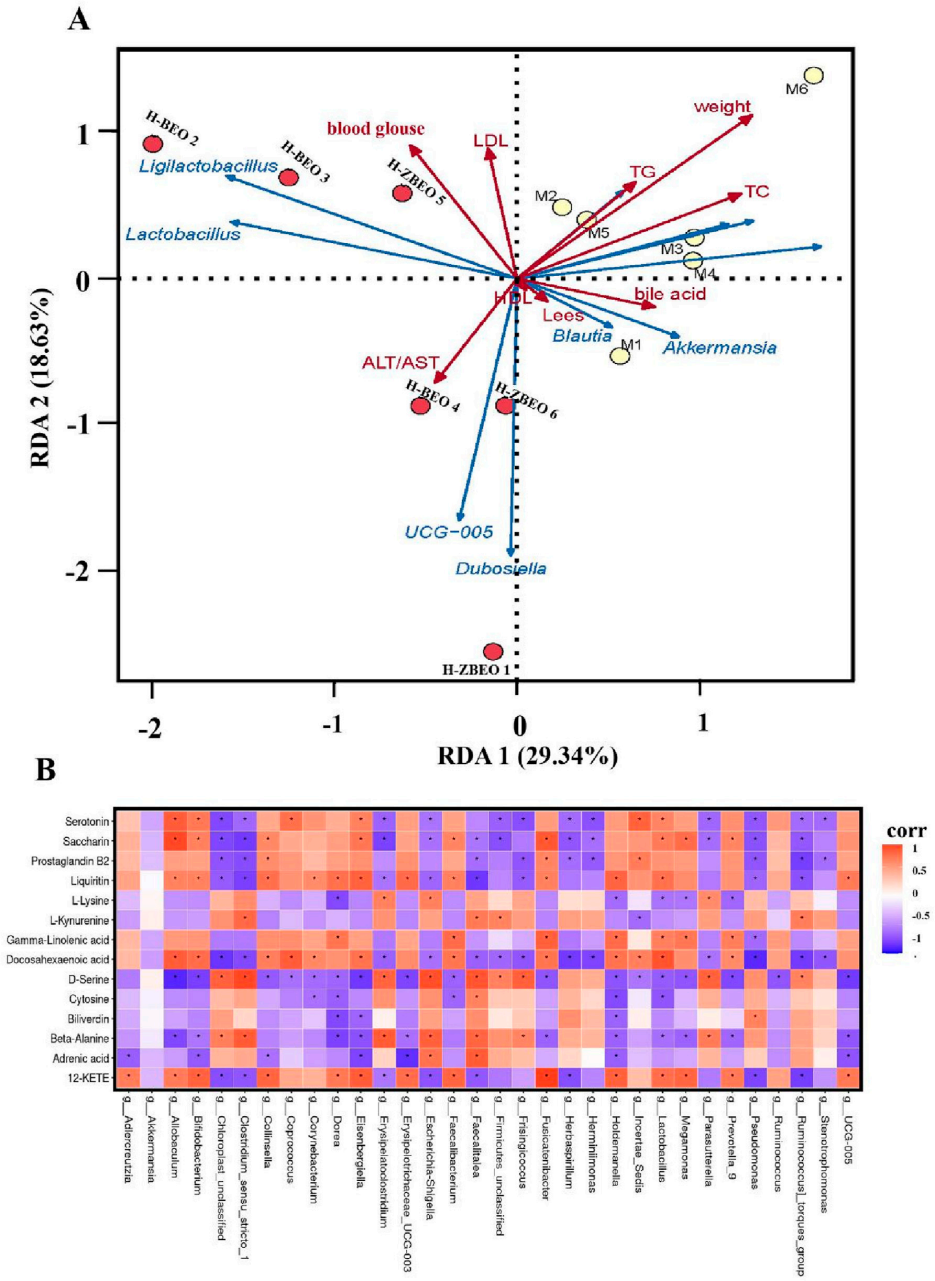
Correlation analysis between gut microbiota and serum biomarkers. **(A)** RDA correlation analysis between environmental factors and gut microbial genus level. **(B)** Heatmap of RDA correlation analysis between serum metabolites and gut microbiota at the genus level.

As shown above, ZBEO treatment significantly altered the composition and abundance of gut microbiota, reduced body weight and serum glucose and lipid levels, and repair liver function in HFD-fed rats. To better understand the role of gut microbiota in the pharmacological effects of ZBEO, Spearman’s correlation analysis was performed to clarify the relationship between gut microbiota and obese biomarkers. The results showed that blood glucose, LDL, and AST/ALT were positively correlated with the relative abundance of *Lactobacillus* and *Ligilactobacillus*, TG, TC, weight, bile acid, and Lees index were positively correlated with the relative abundance of *Akkermansia* and *Blautia*, whereas HDL was negatively correlated with the relative abundance of *Lactobacillus* and *Ligilactobacillus* (Figure 7A). Furthermore, Pearson correlation analysis was used to explore the potential association between gut microbiota genera and serum biomarkers. The correlation between the two is presented in the heat map plot in Figure 7B. Gamma-linolenic acid (GLA) and docosahexaenoic acid (DHA) are key metabolites in the pathway of biosynthesis of unsaturated fatty acids, and DHA was negatively correlated with 13 species and positively correlated with 12 species of bacteria (Figure 7B). Notably, the strongest negative correlation was observed with the *g_Pseudomonas*, while the strongest positive correlation was found with the genus *g_Lactobacillus*. GLA was negatively correlated with *g_Pseudomonas* and positively correlated with *g_Megamonas*, *g_Lactobacillus*, *g_Holdemanella*, *g_Holdemanella*, *g_Fusicatenibacte*r, *g_Faecalitalea* and *g_Dorea*. L-lysine is a key metabolite in the biotin metabolism pathway, and the results in the figure show that L-lysine was negatively correlated with *g_Dorea*, *g_Holdemanella*, *g_Megamonas*, *g_Lactobacillu*s, and *g_Prevotella_9*, and positively correlated with g Erysipelatoclostridium, *g_Escherichia-Shigella* and *g_Parasutterella* positively correlated (Figure 7B). β-alanine, a participant in the pyrimidine metabolic pathway, exhibited a significant inverse relationship with 11 microbial species, while it demonstrated a notable positive correlation with seven other species (Figure 7B). Therefore, we propose that ZBEO regulates obesity by balancing the gut microbiota. The mechanism of ZBEO’s weight-reducing effects may be the result of the interaction between the gut microbiota and metabolic products.

## 4 Discussion

Although ZS and BZ are well-known TCM for improving lipid metabolism, the bioactive ingredients and potential mechanisms of this formula are not fully understood. Given the abundance of volatile constituents within these two medicines, we have employed transdermal delivery to ensure a more convenient application and enhanced absorption. We found that atractylon, D-limonene, and γ-elemene are the main components of ZBEO and decreased the levels of total lipids. Transdermal administration of ZBEO at the abdominal skin effectively reduces food intake and weight in HFD-induced obese rats, improves glucose and lipid metabolism, inhibits appetite-suppressing hormones secretion, and balances the gut microbiota. These results suggest the potential value of ZBEO in the management of obesity and metabolic disorders. It should be noted that due to time constraints and the animal ethical approval process, this study could not be supplemented with experiments on body weight changes and dynamic monitoring of liver and kidney parameters in normal rats. We will systematically analyze the effects of ZBEO on feeding behavior, energy metabolism, and liver and kidney functions in normal animals in subsequent studies.

**FIGURE 8:**
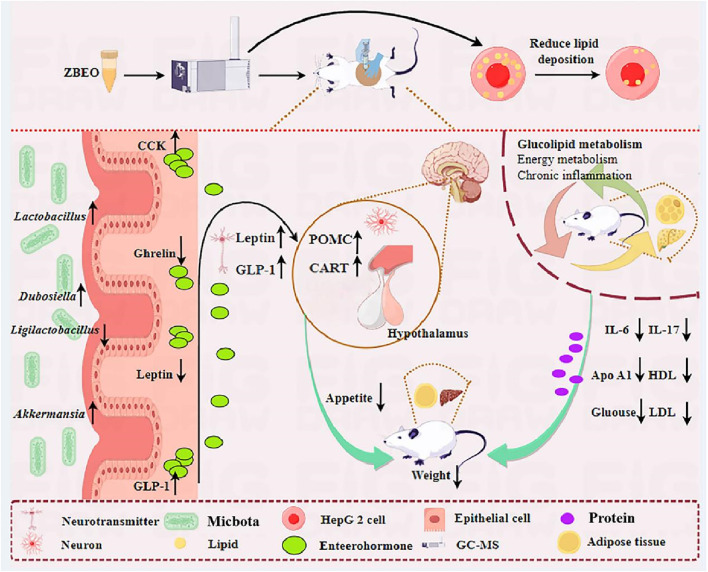
Schema showing the mechanism by which ZBEO exerts its anti-obesity effects. ZBEO: *Citrus aurantium L.-Atractylodes macrocephala Koidz* essential oil.

TCM usually exerts multifunctional synergistic effects through multi-ingredients, multi-targets and multi-pathways. On the one hand, we found that ZBEO significantly reduced the levels of TC, TG, and blood glucose in obese rats, which is consistent with a previous report (Alanazi et al., 2023). On the other hand, ZBEO significantly reduced the content of LDL-c (known as bad cholesterol) and effectively increased the content of HDL-c (known as good cholesterol). In addition, AST and ALT are liver-specific enzymes, and elevated serum AST and ALT activities reflect liver cell damage (On et al., 2023). In our study, H-ZBEO treatment lowered the ALT/AST ratio to a level close to that of the normal group, suggesting improved liver function. Moreover, ZBEO treatment significantly improved the histological changes in the liver and adipose tissue, especially H-ZBEO. Together, ZBEO can alleviate obesity-induced abnormalities in glucose and lipid levels, as well as liver and kidney damage (Figure 8).

GLP-1 and leptin are important hormones that regulate glucose and lipid metabolism, and control energy balance (Nguyen et al., 2023). Imbalance in energy metabolism caused by a HFD is considered a major factor leading to obesity. Previous studies have shown that GLP-1 sends signals of satiety and is crucial for glucose homeostasis, gastrointestinal motility, and appetite (Phung et al., 2022). Similarly, leptin can inhibit fat tissue synthesis, suppress appetite, and reduce energy intake by acting on the hypothalamic metabolic regulation center (Li et al., 2019). Our study showed that ZBEO reduced orexin A, ghrelin, and leptin, while increasing CCK and GLP-1, suggesting that ZBEO may affect energy metabolism and balance by acting on the appetite pathway. The relative expression levels of genes pivotal to feeding behavior and energy homeostasis were further quantified, including AMPK-α, POMC, NPY, and CART. The results showed that ZBEO promoted the expression levels of POMC and CART, both of which are widely recognized as satiety signals involved in appetite suppression. The findings reveal that ZBEO-induced weight loss may be mediated by regulating the organism’s feeding mechanisms, thereby improving energy metabolism in HFD-induced obese rats and improving their feeding behavior (Figure 8).

Metabolomics is instrumental in uncovering the multifaceted targeting and the intricate pathways at play within TCM. In this study, we found significant changes in the metabolic phenotype of obese rats, while the H-ZBEO group was closer to the normal control group and showed partial overlap, indicating a tendency towards normalization of metabolite levels. Moreover, H-ZBEO treatment significantly enhanced unsaturated fatty acid metabolism in obese rats, increasing key metabolites such as DHA, GLA, L-lysine, β-alanine and pyrimidine. Some studies have shown that the metabolism pathway of unsaturated fatty acids can regulate lipid metabolism and reduce serum levels of TC, TG, LDL-c, and glucose (Meyer et al., 2024; Romero-Marco et al., 2024). DHA can reduce inflammatory factors in adipose tissue and benefit lipid metabolism (Yang et al., 2024). GLA is associated with abdominal obesity and can improve metabolic disorders (Shramko et al., 2023). L-lysine administration efficiently prevent the accumulation of TG and TC in the liver and inhibit obesity (Sato et al., 2018; Jalal et al., 2022; Jozi et al., 2022), and β-alanine and pyrimidine play a crucial role in energy expenditure (Nucci et al., 2023). Therefore, ZBEO may improve glucose and lipid metabolism disorders, reduce inflammation, and promote weight loss by regulating changes in these metabolites (Figure 8).

Gut microbiota dysbiosis is closely associated with the development of various pathogenic diseases, including obesity (Lone et al., 2018; Bianchi et al., 2019). Consistent with an elevated *Firmicutes/Bacteroidetes (F/B)* ratio in obesity-related metabolic disorders (Yanez et al., 2021), we found a significant increase in the relative abundance of *Firmicutes* and a significant decrease in the relative abundance of *Bacteroidetes* in the gut microbiota of rats in the model group. Since *Firmicutes* can promote carbohydrate metabolism and energy absorption, ultimately leading to obesity (Li et al., 2022), we detected the microbial community *in vivo* at the genus level. It was found that H-ZBEO increased the relative abundance of *Akkermansia*, *Lactobacillus*, and *Dubosiella*, while decreasing the relative abundance of *Ligilactobacillu*. Previous research has demonstrated that *Akkermansia* can reduce weight gain and fat mass in rats fed with a HFD (Hu et al., 2023); the abundance of *Lactobacillus* is closely related to glucose metabolism, and its abundance is negatively correlated with body weight (Crovesy et al., 2017); the abundance of *Dubosiella* is negatively correlated with weight and positively correlated with the iBAT index (Wang Y. et al., 2022); the abundance of *Ligilactobacillus* increases in obese mice and is positively correlated with the severity of obesity (Huang et al., 2023). In summary, ZBEO exerts its anti-obesity effects by regulating the abundance of the gut microbiota, which in turn modulates glucose and lipid metabolism as well as energy balance (Figure 8).

## 5 Conclusion

In conclusion, atractylon, D-limonene and γ-elemene were the main components of ZBEO. ZBEO and its main components can alleviate disorder of glucose and lipid metabolism, and inflammation *in vivo* and *in vitro*. ZBEO inhibits appetite by regulating AMPK/NPY/POMC/CART pathways, which contributes to decreasing food intake. ZBEO may also alleviates metabolic disorders by regulating biosynthesis of unsaturated fatty acids, biotin metabolism and pyrimidine metabolism pathways, and changes the composition and function of the gut microbiota, which are related to the anti-obesity effect. Overall, these results indicate that ZBEO could be a promising candidate to prevent obesity.

## Data Availability

The raw data supporting the conclusions of this article will be made available by the authors, without undue reservation.

## References

[B1] Ahn Y. J. Maya J. Singhal V. (2024). Update on pediatric anti-obesity medications-current landscape and approach to prescribing. Curr. Obes. Rep. 13 (2), 295–312. 10.1007/s13679-024-00566-z 38689134

[B2] Alanazi S. M. Alsaqer R. A. Alsaeed F. I. Almakhaytah R. M. Buwashl N. T. Mohamed M. E. (2023). Studying the actions of sage and thymoquinone combination on metabolic syndrome induced by high-fat diet in rats. Eur. Rev. Med. Pharmacol. Sci. 27 (6), 2404–2418. 10.26355/eurrev_202303_31775 37013759

[B3] Aukan M. I. Coutinho S. Pedersen S. A. Simpson M. R. Martins C. (2023). Differences in gastrointestinal hormones and appetite ratings between individuals with and without obesity-a systematic review and meta-analysis. Obes. Rev. 24 (2), e13531. 10.1111/obr.13531 36416279 PMC10078575

[B4] Bianchi F. Duque A. Saad S. M. I. Sivieri K. (2019). Gut microbiome approaches to treat obesity in humans. Appl. Microbiol. Biotechnol. 103 (3), 1081–1094. 10.1007/s00253-018-9570-8 30554391

[B5] Bonilla-Carvajal K. Stashenko E. E. Moreno-Castellanos N. (2022). Essential oil of carvone chemotype Lippia alba (Verbenaceae) regulates lipid mobilization and adipogenesis in adipocytes. Curr. Issues Mol. Biol. 44 (11), 5741–5755. 10.3390/cimb44110389 36421673 PMC9688983

[B6] Brahe L. K. Astrup A. Larsen L. H. (2016). Can we prevent obesity-related metabolic diseases by dietary modulation of the gut microbiota? Adv. Nutr. 7 (1), 90–101. 10.3945/an.115.010587 26773017 PMC4717895

[B7] Carrillo-Iregui A. Lopez-Mitnik G. Cossio S. Garibay-Nieto N. Arheart K. L. Messiah S. E. (2010). Relationship between aminotransferases levels and components of the metabolic syndrome among multiethnic adolescents. J. Pediatr. Endocrinol. and Metabolism 23 (12), 1253–1261. 10.1515/jpem.2010.199 21714459

[B8] Chen L. G. Jan Y. S. Tsai P. W. Norimoto H. Michihara S. Murayama C. (2016). Anti-inflammatory and antinociceptive constituents of Atractylodes japonica koidzumi. J. Agric. Food Chem. 64 (11), 2254–2262. 10.1021/acs.jafc.5b05841 26919689

[B9] Crovesy L. Ostrowski M. Ferreira D. Rosado E. L. Soares-Mota M. (2017). Effect of Lactobacillus on body weight and body fat in overweight subjects: a systematic review of Randomized controlled clinical trials. Int. J. Obesuty 41 (11), 1607–1614. 10.1038/ijo.2017.161 28792488

[B10] De Lorenzo A. Gratteri S. Gualtieri P. Cammarano A. Bertucci P. Di Renzo L. (2019). Why primary obesity is a disease. J. Trnaslational Med. 17, 169. 10.1186/s12967-019-1919-y 31118060 PMC6530037

[B11] Fu S. Dang Y. Xu H. Li A. Zhou X. Gao X. (2022). Aloe vera-fermented beverage ameliorates obesity and gut dysbiosis in high-fat-diet mice. Foods 11 (22), 3728. 10.3390/foods11223728 36429320 PMC9689851

[B12] Gioxari A. Amerikanou C. Valsamidou E. Kleftaki S. A. Tzavara C. Kalaitzopoulou A. (2023). Chios mastiha essential oil exhibits antihypertensive, hypolipidemic and anti-obesity effects in metabolically unhealthy adults - a randomized controlled trial. Pharmacol. Res. 194, 106821. 10.1016/j.phrs.2023.106821 37329633

[B13] Guo L. X. Chen G. Yin Z. Y. Zhang Y. H. Zheng X. X. (2019). p-Synephrine exhibits anti-adipogenic activity by activating the Akt/GSK3β signaling pathway in 3T3-L1 adipocytes. J. Food Biochem. 43 (11), e13033. 10.1111/jfbc.13033 31486092

[B14] Hruby A. Hu F. B. (2015). The epidemiology of obesity: a big picture. Pharmaconconomics 33 (7), 673–689. 10.1007/s40273-014-0243-x 25471927 PMC4859313

[B15] Hu Y. He Z. Zhang J. Zhang C. Wang Y. Zhang W. (2023). Effect of Piper nigrum essential oil in dextran sulfate sodium (DSS)-induced colitis and its potential mechanisms. Phytomedicine 119, 155024. 10.1016/j.phymed.2023.155024 37597364

[B16] Huang J. Zhao L. Sun J. Wang L. Gu J. Liu X. (2022). Clinical evidence and potential mechanisms of complementary treatment of ling gui zhu gan formula for the management of serum lipids and obesity. Evidence-Based Complementary Altern. Med. 2022, 7714034. 10.1155/2022/7714034 35586687 PMC9110158

[B17] Huang S. Zou Y. Tang H. Zhuang J. Ye Z. Wei T. (2023). Cordyceps militaris polysaccharides modulate gut microbiota and improve metabolic disorders in mice with diet-induced obesity. J. Sci. Food Agric. 103 (4), 1885–1894. 10.1002/jsfa.12409 36571152

[B18] Jalal K. Khan F. Nawaz S. Afroz R. Khan K. Ali S. B. (2022). Anxiolytic, anti-nociceptive and body weight reducing effects of L-lysine in rats: relationship with brain serotonin an *in-vivo* and in-silico study. Biomed. Pharmacother. 152, 113235. 10.1016/j.biopha.2022.113235 35696944

[B19] Jia S. Gao Z. Yan S. Chen Y. Sun C. Li X. (2016). Anti-obesity and hypoglycemic effects of Poncirus trifoliata L. extracts in high-fat diet C57BL/6 mice. Molecules 21 (4), 453. 10.3390/molecules21040453 27058520 PMC6273343

[B20] Jozi F. Kheiripour N. Taheri M. A. Ardjmand A. Ghavipanjeh G. Nasehi Z. (2022). L-lysine ameliorates diabetic nephropathy in rats with streptozotocin-induced diabetes mellitus. BioMed Res. Int. 2022, 4547312. 10.1155/2022/4547312 36132073 PMC9484891

[B21] Kim H. C. Choi K. S. Jang Y. H. Shin H. W. Kim D. J. (2006). Normal serum aminotransferase levels and the metabolic syndrome: Korean national health and nutrition examination surveys. Yonsei Med. J. 47 (4), 542–550. 10.3349/ymj.2006.47.4.542 16941745 PMC2687736

[B22] Kiso Y. Tohkin M. Hikino H. (1983). Antihepatotoxic principles of Atractylodes rhizomes. J. Nat. Prod. 46 (5), 651–654. 10.1021/np50029a010 6418860

[B23] Lee H. S. Jung J. I. Hwang J. S. Hwang M. O. Kim E. J. (2023). Cydonia oblonga Miller fruit extract exerts an anti-obesity effect in 3T3-L1 adipocytes by activating the AMPK signaling pathway. Nutr. Res. Pract. 17 (6), 1043–1055. 10.4162/nrp.2023.17.6.1043 38053822 PMC10694414

[B24] Li D. Wu H. Dou H. (2019). Weight loss effect of sweet orange essential oil microcapsules on obese SD rats induced by high-fat diet. Biosci. Biotechnol. Biochem. 83 (5), 923–932. 10.1080/09168451.2019.1578640 30741117

[B25] Li F. Yang S. Zhang L. Qiao L. Wang L. He S. (2022). Comparative metagenomics analysis reveals how the diet shapes the gut microbiota in several small mammals. Ecol. Evol. 12 (1), e8470. 10.1002/ece3.8470 35136548 PMC8809447

[B26] Liu X. Y. Fan M. L. Wang H. Y. Yu B. Y. Liu J. H. (2017). Metabolic profile and underlying improved bio-activity of Fructus aurantii immaturus by human intestinal bacteria. Food Funct. 8 (6), 2193–2201. 10.1039/c6fo01851c 28504280

[B27] Lone J. B. Koh W. Y. Parray H. A. Paek W. K. Lim J. Rather I. A. (2018). Gut microbiome: microflora association with obesity and obesity-related comorbidities. Microb. Pathog. 124, 266–271. 10.1016/j.micpath.2018.08.036 30138755

[B28] Marteau J. B. Samara A. Dedoussis G. Pfister M. Visvikis-Siest S. (2009). Candidate gene microarray analysis in peripheral blood cells for studying hypertension/obesity. Pertsonalized Med. 6 (3), 269–291. 10.2217/pme.09.6 29783504

[B29] Meyer N. M. T. Pohrt A. Wernicke C. Pletsch-Borba L. Apostolopoulou K. Haberbosch L. (2024). Improvement in visceral adipose tissue and LDL cholesterol by high PUFA intake: 1-year results of the nutriact trial. Nutrients 16 (7), 1057. 10.3390/nu16071057 38613089 PMC11013849

[B30] Nguyen N. P. K. Tran K. N. Nguyen L. T. H. Shin H. M. Yang I. J. (2023). Effects of essential oils and fragrant compounds on appetite: a systematic review. Int. J. Mol. Sci. 24 (9), 7962. 10.3390/ijms24097962 37175666 PMC10178777

[B31] Nie M. Z. Liu Y. Z. Chen H. Z. Huang S. C. Liu L. H. (2022). Efficacy of the addition and subtraction of Hovenia allium cinnamon soup on stable coronary heart disease and its effect of serum VEGE, ET-1 and CRP. Drug Eval. 19 (05), 293–295. 10.19939/i.cnki.1672-2809.2022.05.10

[B32] Nucci R. a. B. Filho V. a. N. Jacob-Filho W. Otoch J. P. Pessoa A. F. M. (2023). Role of nutritional supplements on gut-muscle axis across age: a mini-review. Cell Physiol. Biochem. 57 (3), 161–168. 10.33594/000000628 37190847

[B33] On J. Y. Kim S. H. Kim J. M. Park S. Kim K. H. Lee C. H. (2023). Effects of fermented Artemisia annua L. and Salicornia herbacea L. on inhibition of obesity *in vitro* and in mice. Nutrients 15 (9), 2022. 10.3390/nu15092022 37432154 PMC10180564

[B34] Paturi G. Butts C. A. Stoklosinski H. Herath T. D. Monro J. A. (2017). Short-term feeding of fermentable dietary fibres influences the gut microbiota composition and metabolic activity in rats. Int. J. Food Sci. and Technol. 52 (12), 2572–2581. 10.1111/ijfs.13543

[B35] Phung H. M. Jang D. Trinh T. A. Lee D. Nguyen Q. N. Kim C. E. (2022). Regulation of appetite-related neuropeptides by Panax ginseng: a novel approach for obesity treatment. J. Ginseng Res. 46 (4), 609–619. 10.1016/j.jgr.2022.03.007 35818423 PMC9270656

[B36] Romero-Marco P. Chicharro C. Verde Z. Miguel-Tobal F. Fernandez-Araque A. (2024). Effect on blood lipids and body composition of a high-fat (MUFA) and high-fiber diet: a case-control study. Food Sci. and Nutr. 12 (6), 3863–3871. 10.1002/fsn3.4042 38873480 PMC11167160

[B37] Saltiel A. R. Olefsky J. M. (2017). Inflammatory mechanisms linking obesity and metabolic disease. J. Clin. Investigation 127 (1), 1–4. 10.1172/JCI92035 28045402 PMC5199709

[B38] Sato T. Muramatsu N. Ito Y. Yamamoto Y. Nagasawa T. (2018). L-lysine attenuates hepatic steatosis in senescence-accelerated mouse prone 8 mice. J. Nutr. Sci. Vitaminology 64 (3), 192–199. 10.3177/jnsv.64.192 29962430

[B39] Shramko V. S. Shcherbakova L. V. Kashtanova E. V. Stakhneva E. M. Polonskaya Y. V. Ragino Y. I. (2023). Associations of fatty acid profile with abdominal obesity in men. Bull. Exp. Biol. Med. 175 (5), 629–632. 10.1007/s10517-023-05915-x 37861907

[B40] Sousa-Ferreira L. De Almeida L. P. Cavadas C. (2014). Role of hypothalamic neurogenesis in feeding regulation. Trends Endocrinol. and Metabolism 25 (2), 80–88. 10.1016/j.tem.2013.10.005 24231724

[B41] Su J. Guo Q. Cai Y. Wang T. Mao L. Gao Y. (2020). Effect of Ultra-high temperature processing on the physicochemical properties and antibacterial activity of d-limonene emulsions stabilized by β-lactoglobulin/Gum Arabic bilayer membranes. Food Chem. 332, 127391. 10.1016/j.foodchem.2020.127391 32603920

[B42] Tang C. Wang Y. Chen D. Zhang M. Xu J. Xu C. (2023). Natural polysaccharides protect against diet-induced obesity by improving lipid metabolism and regulating the immune system. Food Res. Int. 172, 113192. 10.1016/j.foodres.2023.113192 37689942

[B43] Terzo S. Calvi P. Nuzzo D. Picone P. Allegra M. Mule F. (2023). Long-term ingestion of Sicilian black bee chestnut honey and/or D-limonene counteracts brain damage induced by high fat-diet in obese mice. Int. J. Mol. Sci. 24 (4), 3467. 10.3390/ijms24043467 36834882 PMC9966634

[B44] Uemura H. Katsuura-Kamano S. Yamaguchi M. Sawachika F. Arisawa K. (2014). Serum hepatic enzyme activity and alcohol drinking status in relation to the prevalence of metabolic syndrome in the general Japanese population. Plos One 9 (4), e95981. 10.1371/journal.pone.0095981 24755715 PMC3995980

[B45] Wang J. Feng W. Zhang S. Chen L. Sheng Y. Tang F. (2019). Ameliorative effect of Atractylodes macrocephala essential oil combined with Panax ginseng total saponins on 5-fluorouracil induced diarrhea is associated with gut microbial modulation. J. Ethnopharmacol. 238, 111887. 10.1016/j.jep.2019.111887 31004726

[B46] Wang L. Wang F. Zhang X. Chen Q. Xu J. Li H. (2022a). Transdermal administration of volatile oil from Citrus aurantium - rhizoma atractylodis macrocephalae alleviates constipation in rats by altering host metabolome and intestinal microbiota composition. Oxidative Med. Cell. Longev. 2022, 9965334. 10.1155/2022/9965334 35087623 PMC8789429

[B47] Wang L. F. Liu X. L. Li H. T. Chen Q. Y. Wang Y. Zou B. (2020). Mechanism of Aurantii fructus immaturus volatile oil in treatment of slow transit constipation based on network pharmacology. China J. Chin. Materia Medica 45 (8), 1909–1917. 10.19540/j.cnki.cjcmm.20200207.302 32489077

[B48] Wang Y. Li T. Liu Y. Yang C. Liu L. Zhang X. (2022b). Heimao tea polysaccharides ameliorate obesity by enhancing gut microbiota-dependent adipocytes thermogenesis in mice fed with high fat diet. Food Funct. 13 (24), 13014–13027. 10.1039/d2fo02415b 36449351

[B49] Yanez C. M. Hernandez A. M. Sandoval A. M. Dominguez M. a. M. Muniz S. a. Z. Gomez J. O. G. (2021). Prevalence of Blastocystis and its association with Firmicutes/Bacteroidetes ratio in clinically healthy and metabolically ill subjects. BMC Microbiol. 21 (1), 339. 10.1186/s12866-021-02402-z 34895145 PMC8665487

[B50] Yang X. Li X. Hu M. Huang J. Yu S. Zeng H. (2024). EPA and DHA differentially improve insulin resistance by reducing adipose tissue inflammation-targeting GPR120/PPARγ pathway. J. Nutr. Biochem. 130, 109648. 10.1016/j.jnutbio.2024.109648 38631512

[B51] Yuri G. Sanhueza S. Paredes A. Morales G. Cifuentes M. Ormazabal P. (2023). Deleterious liver-adipose crosstalk in obesity: hydroethanolic extract of Lampaya medicinalis Phil. (Verbenaceae) counteracts fatty acid-induced fibrotic marker expression in human hepatocytes. Mol. Cell. Endocrinol. 564, 111882. 10.1016/j.mce.2023.111882 36736687

[B52] Zhong Q. Xiao X. Qiu Y. Xu Z. Chen C. Chong B. (2023). Protein posttranslational modifications in health and diseases: functions, regulatory mechanisms, and therapeutic implications. MedComm (2020) 4 (3), e261. 10.1002/mco2.261 37143582 PMC10152985

[B53] Zhu B. Zhang Q. L. Hua J. W. Cheng W. L. Qin L. P. (2018). The traditional uses, phytochemistry, and pharmacology of Atractylodes macrocephala Koidz.: a review. J. Ethnopharmacol. 226, 143–167. 10.1016/j.jep.2018.08.023 30130541

[B54] Zou J. Chassaing B. Singh V. Pellizzon M. Ricci M. Fythe M. D. (2018). Fiber-mediated nourishment of gut microbiota protects against diet -induced obesity by restoring IL-22-mediated colonic health. Cell Host and Microbe 23 (1), 41–53. 10.1016/j.chom.2017.11.003 29276170 PMC6005180

