# Correction: Transdermal administration of herbal essential oil alleviates high-fat diet-induced obesity by regulating metabolism and gut microbiota

**DOI:** 10.3389/fphar.2025.1743063

**Published:** 2025-12-05

**Authors:** Zu-Wen Ye, Qi-Yue Yang, Dong-Hua Yang, Qiao-Hong Lin, Xiao-Xia Liu, Feng-Qin Li, Fang-Fang Yan, Ping Luo, Si Qin, Fang Wang

**Affiliations:** 1 Cancer Research Center, The Jiangxi Province Key Laboratory for Diagnosis, Treatment, and Rehabilitation of Cancer in Chinese Medicine, Jiangxi University of Chinese Medicine, Nanchang, China; 2 Department of Endocrinology, Hospital of Chengdu University of Traditional Chinese Medicine, Chengdu, China; 3 New York College of Traditional Chinese Medicine, Minola, NY, United States; 4 Lab of Food Function and Nutrigenomics, College of Food Science and Technology, Hunan Agricultural University, Changsha, China

**Keywords:** obesity, essential oil, lipid mechanism, inflammation, gut microbiota

**Affiliation 2** was erroneously listed as “Affiliated Hospital of Chengdu University of Traditional Chinese Medicine, Chengdu, China”. This was an error, and the affiliation has subsequently been removed.

**Affiliation 2** “Department of Endocrinology, Hospital of Chengdu University of Traditional Chinese Medicine, Chengdu, China” was omitted from the paper. This affiliation has now been added for author **Qi-Yue Yang**.

Author **Fang-Fang Yan** was erroneously assigned to “Affiliated Hospital of Chengdu University of Traditional Chinese Medicine, Chengdu, China”. The author is assigned to **Affiliation 4**.

The original article has been updated.

