# Supplementary material for: Transdermal administration of herbal essential oil alleviates high-fat diet-induced obesity by regulating metabolism and gut microbiota
Source: Front Pharmacol. 2025 Mar 19;16:1565030. doi: 10.3389/fphar.2025.1565030 (PMC11962428; doi:10.3389/fphar.2025.1565030)
Supplement: Supplementary file 1 [file DataSheet1.docx]

Supplementary Material

# Supplementary Figures and Tables

## Supplementary Figures


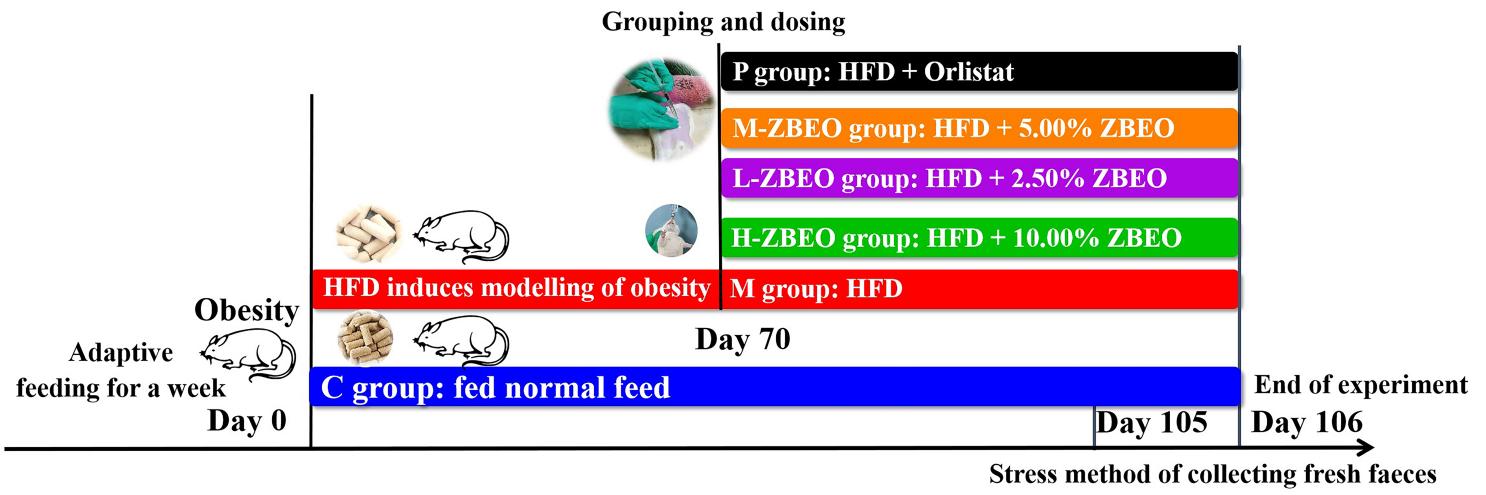


**Supplementary Figure 1.** Rat experiment processing.


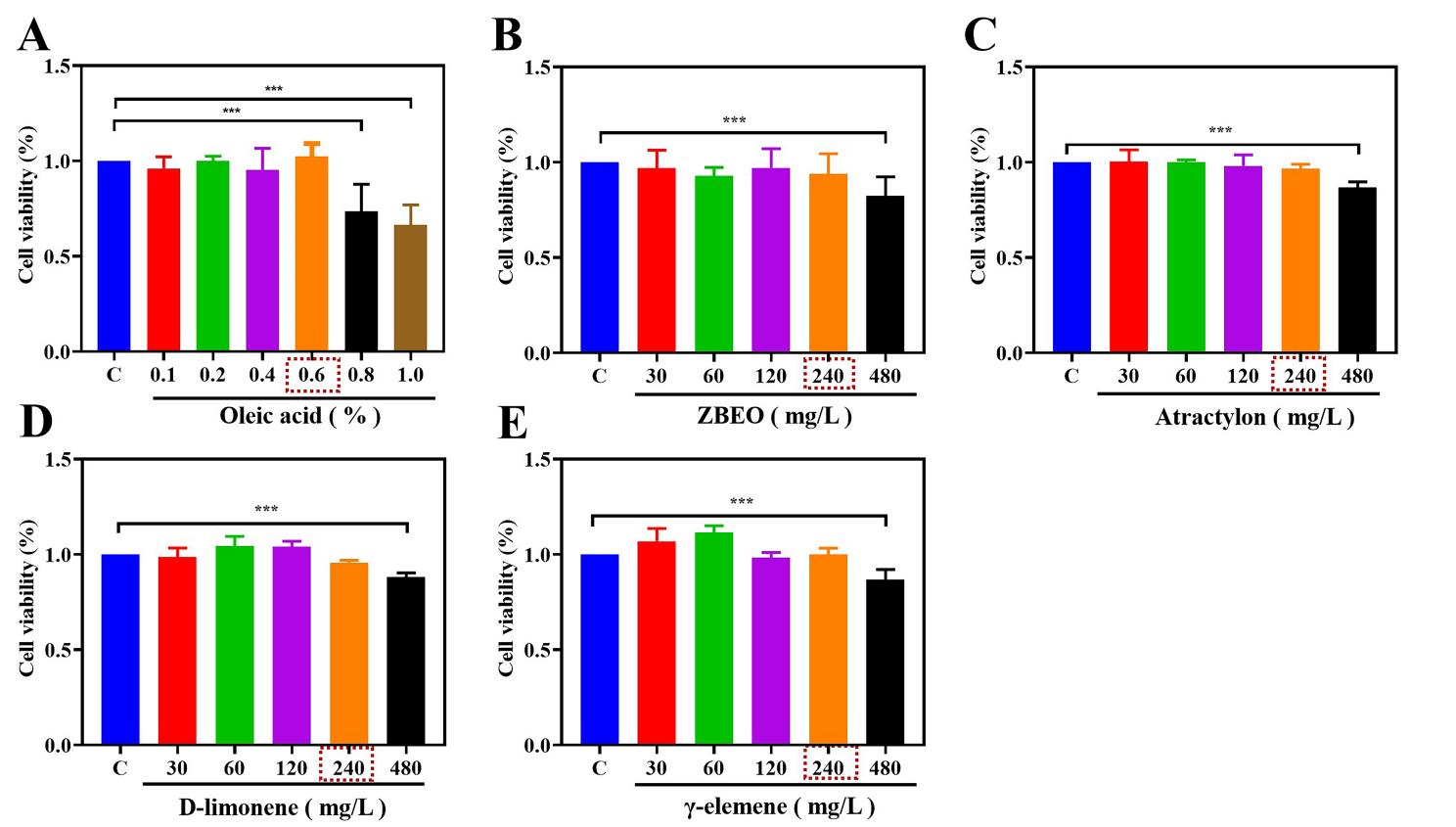


**Supplementary Figure 2.** The effect of oleic acid, ZBEO and its main components on the activity of HepG2 cells were detected by CCK-8 assay. (A) Oleic acid. (B) ZBEO. (C) atractylon. (D) D-limonene. (e) γ-elemene.


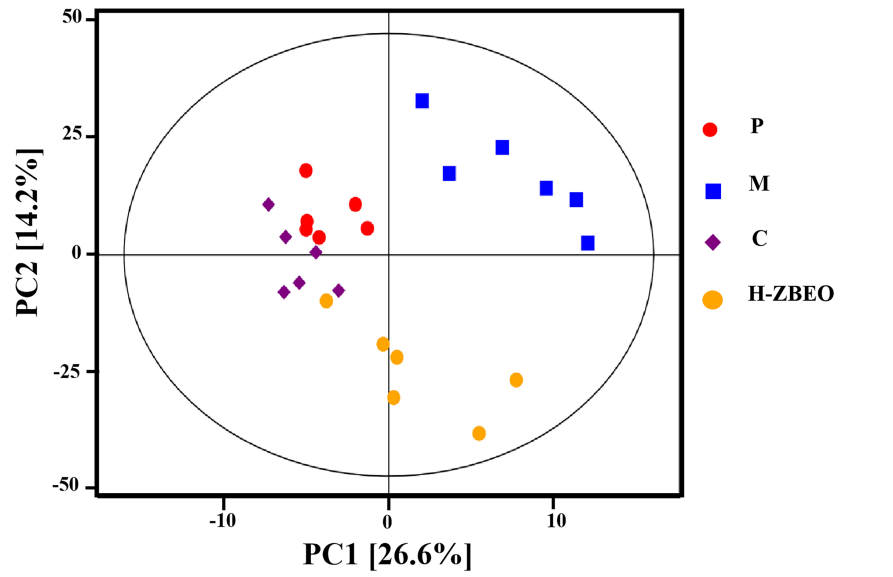


**Supplementary Figure 3.** Effects of ZBEO on serum metabolite profiles. Score plots of PCA.

## Supplementary Tables

**Supplementary Table 1** Compounds detected in ZBEO by GC–MS. Data was expressed in relative percentages of area values (%).

| **No.** | **Compound Name** | **Molwt** | **Formula** | **Relative**  **Content (%)** |
| --- | --- | --- | --- | --- |
| 1 | Atractylon | 216.32 | C_15_H_20_O | 40.82 |
| 2 | D-limonene | 136.23 | C_10_H_16_ | 8.98 |
| 3 | γ-elemene | 204.35 | C_15_H_24_ | 7.02 |
| 4 | β-selinene | 204.35 | C_15_H_24_ | 4.05 |
| 5 | Trans-caryophyllene | 204.35 | C_15_H_24_ | 1.48 |
| 6 | (-)-cyperene | 204.35 | C_15_H_24_ | 1.30 |
| 7 | α-curcumene | 202.34 | C_15_H_22_ | 0.95 |
| 8 | β-pinene | 136.23 | C_10_H_16_ | 0.82 |
| 9 | β-caryophyllene | 204.35 | C_15_H_24_ | 0.54 |
| 10 | (-)-α-cedrene | 204.35 | C_15_H_24_ | 0.49 |
| 11 | β-sesquiphellandrene | 204.35 | C_15_H_24_ | 0.40 |
| 12 | Atractylenolide Ⅱ | 248.33 | C_15_H_20_O_3_ | 0.39 |
| 13 | β-maaliene | 204.35 | C_15_H_24_ | 0.36 |
| 14 | Germacrene D | 204.35 | C_15_H_24_ | 0.30 |
| 15 | 3-carene | 136.23 | C_10_H_16_ | 0.35 |
| 16 | Farnesene | 204.35 | C_15_H_24_ | 0.21 |
| 17 | Alloaromadendrene | 204.35 | C_15_H_24_ | 0.18 |
| 18 | Valencene | 204.35 | C_15_H_24_ | 0.15 |
| 19 | δ-elemene | 204.35 | C_15_H_24_ | 0.11 |
| 20 | Eugenol | 164.20 | C_10_H_12_O_2_ | 0.09 |
| 21 | Palmitic acid | 256.42 | C_16_H_32_O_2_ | 0.08 |
| 22 | (-)-isoledene | 204.35 | C_15_H_24_ | 0.08 |
| 23 | α-pinene | 136.23 | C_10_H_16_ | 0.07 |
| 24 | (+)-δ-Cadinene | 204.35 | C_15_H_24_ | 0.07 |
| 25 | (-)-spathulenol | 220.35 | C_15_H_24_O | 0.07 |
| 26 | D-carvone | 150.22 | C_10_H_14_O | 0.07 |
| 27 | Methyl-4-(1,5-dimethyl-1-hydroxy-  4(5)-hexenyl)-1-cyclohexene | 222.37 | C_15_H_26_O | 0.06 |
| 28 | Methyleugenol | 178.228 | C_11_H_14_O_2_ | 0.06 |
| 29 | 1,2,3,3a,4,7,8,8a-octahydro-1,4,6  -trimethyl-4,7-ethanoazulene | 204.35 | C_15_H_24_ | 0.06 |
| 30 | Terpinolene | 136.23 | C_10_H_16_ | 0.05 |
| 31 | 1,2,3,4,6,8alpha-hexahydro-1-  isopropyl-4,7-dimethylnaphthalene | 204.35 | C_15_H_24_ | 0.04 |
| 32 | 1,3-dimethoxy-5-methylbenzene | 152.19 | C_9_H_12_O_2_ | 0.03 |
| 33 | Cyclofenchene | 136.23 | C_10_H_16_ | 0.03 |
| 34 | (-)-α-gurjunene | 204.35 | C_15_H_24_ | 0.03 |
| 35 | (Z)-β-ocimene | 136.23 | C_10_H_16_ | 0.14 |
| 36 | Butyl acrylate | 128.17 | C_7_H_12_O_2_ | 0.06 |
| 37 | (-)-aristolene | 204.35 | C_15_H_24_ | 0.04 |
| 38 | Octadec-1-ene | 252.48 | C_18_H_36_ | 0.04 |
| 39 | (1R,8aR)-1,2,3,4,6,8a-hexahydro-1,8a-dimethyl-7-(1-methyleneethyl) naphthalene | 202.34 | C_15_H_22_ | 2.23 |
| 41 | Zierone | 218.34 | C_15_H_22_O | 1.51 |
| 42 | Berkheyaradulene | 204.35 | C_15_H_24_ | 0.75 |
| 43 | Selina-3,7(11)-diene | 204.35 | C_15_H_24_ | 0.52 |
| 44 | (1R,8aS)-1,2,6,7,8,8a-hexahydro-1,8a-dimethyl-7-isopropylidenenaphthalene | 202.34 | C_15_H_22_ | 0.99 |
| 45 | Naphthalene,1,2,4a,5,8,8a-hexahydro-4,7-dimethyl-1-(1-methylethyl)-, (1R,4aS,8aR)-rel- | 204.35 | C_15_H_24_ | 0.20 |
| 46 | Anthracene,1,2,3,4,5,6,7,8  -octahydro-9-methyl | 200.32 | C_15_H_20_ | 0.11 |
